# Synthesis, Biological and In Silico Evaluation of Pure Nucleobase-Containing Spiro (Indane-Isoxazolidine) Derivatives as Potential Inhibitors of MDM2–p53 Interaction

**DOI:** 10.3390/molecules24162909

**Published:** 2019-08-10

**Authors:** Loredana Maiuolo, Vincenzo Algieri, Beatrice Russo, Matteo Antonio Tallarida, Monica Nardi, Maria Luisa Di Gioia, Zahra Merchant, Pedro Merino, Ignacio Delso, Antonio De Nino

**Affiliations:** 1 Dipartimento di Chimica e Tecnologie Chimiche, Via P. Bucci, cubo 12C, Università della Calabria, 87036 Rende (CS), Italy; 2 Dipartimento di Scienze della Salute, Università Magna Græcia, Viale Europa, Germaneto, 88100 Catanzaro, Italy; 3 Dipartimento di Farmacia e Scienze della Salute e della Nutrizione, Edificio Polifunzionale, Università della Calabria, 87036 Rende (CS), Italy; 4 Department of Pharmaceutics, UCL School of Pharmacy, London NW14NS, UK; 5 Unidad de Glicobiología, Instituto de Biocomputación y Física de Sistemas Complejos (BIFI), Universidad de Zaragoza, 50009 Zaragoza, Spain; 6 Departamento de Síntesis y Estructura de Biomoléculas, Instituto de Síntesis Química y Catálisis Homogénea (ISQCH), Universidad de Zaragoza-CSIC, 50009 Zaragoza, Spain

**Keywords:** spirooxindoles, isoxazolidines, MDM2–p53 inhibition, indane derivatives

## Abstract

Nucleobase-containing isoxazolidines spiro-bonded to an indane core have been synthesized in very good yields by regio- and diastereoselective 1,3-dipolar cycloaddition starting from indanyl nitrones and *N*-vinylnucleobases by using environmentally benign microwave technology. The contemporary presence of various structural groups that are individually active scaffolds of different typology of drugs, has directed us to speculate that these compounds may act as inhibitors of MDM2–p53 interaction. Therefore, both computational calculations and antiproliferative screening against A549 human lung adenocarcinoma cells and human SH-SY5Y neuroblastoma cells were carried out to support this hypothesis.

## 1. Introduction

Spirocyclic compounds exhibit important bioactivity principally as antimicrobial [1,2], anticancer [3,4] and anti-inflammatory [5] agents. Their therapeutic properties appear to be due to the tetrahedral nature of the spiro carbon that makes the two linked rings more or less perpendicular to each other [6]. In fact, the asymmetric properties of substrates due to chiral spiro center seem to be an important factor affecting biological activities. In particular, spirooxindoles are characterized by a variety of rings spiro-bonded to the C3 of the oxindole core with a variable degree of substitution, which is a scaffold present in many natural products and pharmacologically relevant drugs. [7,8,9,10]

**Figure 1:**
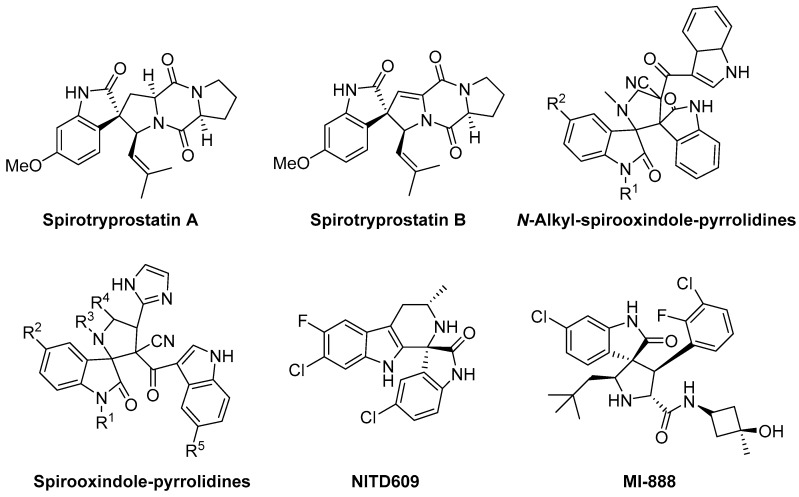
Structure of some representative active spirooxindole derivatives.

Recently, the spirocyclic oxindoles have particularly emerged for their antiviral and anticancer activity [11,12,13]. Some representative examples of natural products or pharmaceutical drugs having a spirooxindole scaffold are illustrated in Figure 1.

In particular, among the various examples, spirooxindole pyrrolidines with a long carbon chain or imidazole/thiazolidine ring have emerged for their anticancer properties against A549 human lung adenocarcinoma cell line [14]. Moreover, NITD609 [15] and MI-888 [16] are currently in preclinical evaluation as inhibitors of malaria and some human cancer cell lines, respectively. More specifically, MI-888 seems to be a potent inhibitor of MDM2–p53 interaction, capable of achieving complete regression of two types of human cancers through oral administration, such as SJSA-1 osteosarcoma and RS4;11 acute lymphoblastic leukaemia tumour xenograft models. In fact, it is now widely documented that the protein p53 is a tumour suppressor and its activity consists principally in preventing tumour formation, in repair of damaged cells or apoptosis to eliminate irreparable cells. However, in several cancers, p53 is effectively inhibited by MDM2 oncoprotein, which is generally responsible for regulation of p53 production. [17,18,19,20,21,22] Therefore, the disruption of the MDM2–p53 interaction is recently recognized as the promising strategy for anticancer drug design. [23,24,25,26,27,28,29]

Inspired by those results, a number of methods to synthesize oxindole derivatives were realized during the last years [30]. The 1,3-dipolar cycloaddition between an oxindole nitrone and an unsaturated substrate can be considered the common procedure for the preparation of spirooxindole structures or spiroisoxazolidines spiro-bonded to an oxindole scaffold, [31,32] although other synthetic strategies are employed including oxidative rearrangement, [33] Heck reaction, [34] intramolecular Mannich reaction, [35] ring expansion [36].

Our group, with wide expertise of Mw-assisted reactions such as 1,3-dipolar cycloadditions [37,38,39,40,41], has recently synthesized a series of isatinyl/indanyl nitrones (INs) by a solvent-free condensation of alkyl- or arylhydroxylamines hydrochlorides with isatin and indanone using a microwave irradiation technique [42]. This methodology can be considered a versatile procedure to synthesize nitrones in high yields and a short reaction time, reducing the formation of by-products. These substrates have demonstrated high reactivity in successive 1,3-dipolar cycloadditions with *N*-vinylnucleobases to produce spiro-isoxazolidines with oxindole-like core and nucleobase.

Herein, we describe the synthesis of some diastereomerically pure nucleobase-containing spiro-(indane isoxazolidine) derivatives as potential anti-cancer substrates, supported by a computational study about a possible inhibition of MDM2–p53 interaction and by their antiproliferative evaluation against A549 human lung carcinoma cells and human SH-SY5Y neuroblastoma cells. In fact, to our knowledge, there are no spiro-compounds active against A549 and SH-SY5Y cells as inhibitors of MDM2–p53 interactions, with structural characteristics as our substrates, which are distinguished by the simultaneous presence of a spiro-carbon, an isoxazolidine portion spiro-bonded to C1 of an indane ring and a nucleobase, in attempt to significantly increase their inhibitory and antiproliferative activities. Moreover, one of the investigations that we desire to highlight is that, apart from the isoxazolidine and nucleobase portion, the indane ring might be an alternative candidate as key moiety, instead of oxindole core of active spiro-substrates. On the other hand, substituted 1-indanones or indan-1,3-diones have proven to be potent cytotoxic agents effective against a wide range of diseases. [43,44] Inter alia, a number of derivatives were active against growth of solid tumors e.g., colon, lung bronchogenic and osteosarcoma for which few drugs are actually available [45].

## 2. Results and Discussion

### 2.1. Chemistry

**Scheme 1:**
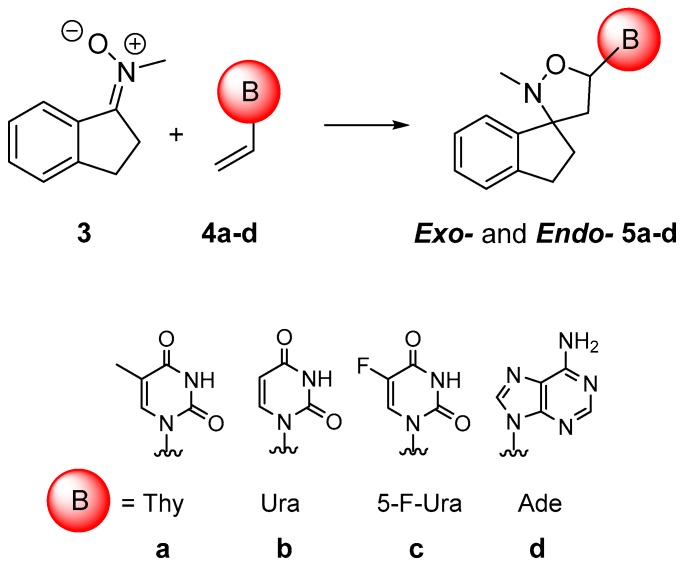
Synthetic route to cycloadducts **5a**–**d**. *Reagents and conditions*: nitrone (2 equiv.), *N*-vinylnucleobase (1 equiv.), 750 W.

The spiro (indane-isoxazolidine) derivatives **5a**–**d** were prepared by MW-assisted 1,3-dipolar cycloaddition reaction between *N*-vinylnucleobases **4a**–**d [46]** and indanyl nitrone **3**, which was synthesized from 1-indanone **1** and *N*-methyl hydroxylamine hydrochloride **2** using our previously reported procedure [42] (Scheme 1).

The *N*-vinyl nucleobases were synthesized by a convenient and *one-pot* procedure of nucleobase vinylation developed by our group, avoiding the tedious and *multi-step* synthetic procedures previously used [46]. The choice of using nucleobases as one of the bio-active scaffold of our compounds was oriented towards the possibility to improve the interactions with the active site of the enzymes involved in this work. With respect to the use of simple aromatic rings as a phenyl group, the nucleobase cycles can form further interactions, such as hydrogen bonds with their amino or carbonyl groups, in addition to π–π interactions of aromatics systems. In the literature, nucleobases such as antimetabolites of p53 are known, in particular, 5-*F*-Ura and purine derivatives [47,48,49]. However, scope of this work was not to synthesize nucleobase derivatives as antimetabolites of p53, but only improve substrate interactions with enzymes through their presence on the synthesized molecule backbone, as previously explicated. On the other hand, during the computational studies [see subsequent section], we have not noticed any of their particular contributions to link them to the characteristics of p53 antimetabolites.

The synthetic procedure consists of the co-grinding of ketonitrone and *N*-vinylnucleobase in a mortar, followed by transfer of the mixture in an appropriate vessel. Then, after further mixing of the solids in a vortex without use of solvents, the solid mixture was placed in a microwave oven for the indicated time. Based on our experience, the grinding phase is important for the reaction trend. On the other hand, in the literature, it is evident that solid-state chemical reactions among particles can take place if the latter are small enough to have adequate Surface-to-Volume ratios. In this situation, the reaction can be carried out in short times and with quantitative formation of products, by the (interfacial) dynamical processes normally taking place in the solid-state, which are further accelerated by microwave irradiation. [50,51,52].

The reaction conditions and results are reported in Table 1.

The optimized reaction conditions were obtained after various attempts, varying power of MW, temperature and ratio nitrone/nucleobase. All compounds were isolated in high yields and very short reaction times if compared with those obtained by classical cycloaddition conditions (toluene, 110 °C, 3 days; yield 20–30%). Cycloaddition reactions showed a total regioselectivity and moderate diasteroselectivity, furnishing the 5-substituted regioisomer, as confirmed by ^1^H-NMR and ^13^C-NMR and a prevalent amount of *exo*-adduct (Table 1). Configuration of the obtained products **5a**–**d** was confirmed by NOESY NMR experiments, after purification by flash-chromatography and HPLC. Moreover, the results obtained in terms of the yield and stereoselectivity of the reaction do not seem to be affected by the pyrimidine or purine nature of the nucleobase.

Because no officially approved biochemical mechanism of inhibition is known for various cancer lines, as a first approach, we performed computational studies on our substrates **5a**–**d** to focus mainly on their behaviour as inhibitors of the MDM2–p53 interaction and to study their possible anti-proliferative action.

### 2.2. Computational Studies

As explained in the introduction, and demonstrated in many recent works, these type of spiro-derivatives are able to disrupt the interaction between MDM2 and p53, which is the origin of their anti-proliferative action [53,54]. Therefore, we performed computational studies on our substrates **5a**–**d** to focus mainly on their behaviour as disruptors of MDM2–p53 complex and to predict their activity.

MDM2–p53 interaction has been widely explored and has been fully described. p53 binds through its subdomain TAD1 (part of the *N*-terminal transactivation domain, TAD), involving residues 17–29, a hydrophobic cleft in the *N*-terminal of MDM2 (aa 25–109). [55,56] Three key residues in p53 are involved in stabilising the interaction: Phe19, Trp23 and Leu26. Although TAD is an intrinsically disordered domain, TAD1 folds to an alpha-helix when it binds to MDM2, fitting perfectly on the cleft [57].

Previous docking studies with different compounds capable to inhibit the MDM2–p53 interaction showed that those compounds are able to mimic the key p53-residues when binding to MDM2 [58,59].

However, very few authors have addressed this system with molecular dynamic simulations, and only the interaction MDM2-ligand was considered [60,61]. Here, we are considering also p53 in order to simulate the whole biological system. We have performed molecular docking calculations with all the synthetized products against MDM2, using Glide and PDB 1YCR as MDM2–p53 structure, deleting the p53 moiety. Five derivatives (***exo*-5a**, ***exo*-5b**, ***exo*-5c**, ***endo*-5c** and ***exo*-5d**) and the corresponding enantiomers were evaluated.

In all cases, low binding energies were obtained, very close among most of the ligands (Table S3). Those poses of minimum energy place the ligand where Phe19 and Trp23 bind to MDM2 (Figure S1A). Remarkably**, *exo-(3R,5R)*-5a** is the only ligand with a pose placing the indane moiety overlapping Trp23 when superimposing both structures (Figure S1B).

Unfortunately, these small energy differences are not enough for justifying the biological activity for **5a**–**d** derivatives, due to the complexity and dynamics of the real system, so p53 must be considered in the interaction, as well as the flexibility of both proteins.

Two type of systems were simulated with molecular dynamics, spiro-isoxazolidines docked to MDM2 as well as **5a**–**5d** bounded to MDM2 in the presence of p53 (Figure S2). For simulating the tertiary complex, two starting structures were considered. For the first one, ligands were docked to the p53–MDM2 complex. For the second starting structure, we firstly manually moved the p53 fragment from the MDM2 hydrophobic cleft, to a distance of 8 Å, and then ligands were docked into the space between p53 and MDM2. MDs were performed with Amber 16 along at least 500 ns for each simulation. Molecular dynamics containing exclusively MDM2 and spiro-derivatives did not show any variation along the simulations, keeping the ligand in the p53 key residues interaction zone, and no significant changes were observed in the protein structure.

Analogously, tertiary systems starting from the complex MDM2–p53 remained stable along the simulations. Ligand surrounds p53 for 50 ns, not being able to disrupt the interaction between the two proteins. After 200 ns, all interaction with MDM2 is lost.

Finally, MD simulations were carried out over the structures containing docked ligands in the hydrophobic cleft of MDM2 in presence of p53. As reference, we also simulated the same system without a ligand. In this reference simulation, TAD1 subdomain is able to bind MDM2 in the hydrophobic groove, folding to a helix in 300 ns of the simulation, remaining completely stable for 200 ns more (Figure S3).

However, the presence of any ligand in the MDM2 cleft prevents p53 to bind as if there was no ligand, and is not able to expel completely the spiro-isoxazolidine. p53 only displaces it, and they both must share the hydrophobic groove, and in most of the simulations, TAD1 is able to fold back to a helix reaching a stable stationary situation.

**Figure 2:**
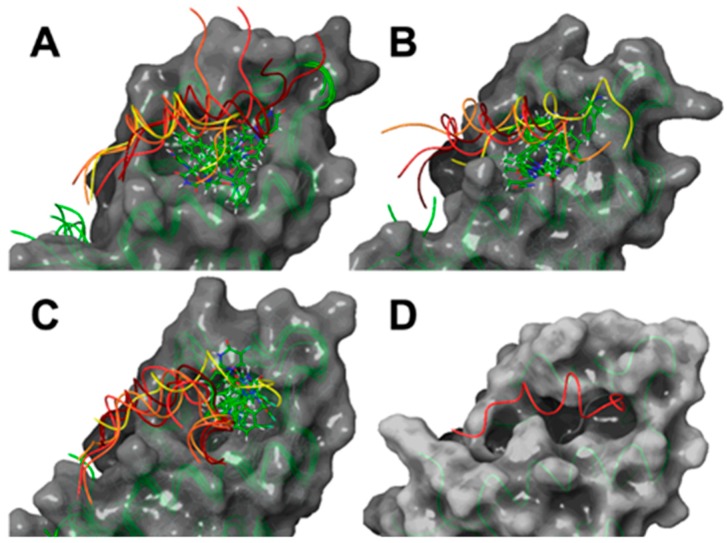
Snapshots from MD simulations. MDM2 in green, p53 from yellow (starting) to dark red (500 ns) containing ***exo*-5b** (**A**); ***endo*-5a** (**B**); ***exo*-5c** (**C**); crystallographic p53-MDM2 complex (**D**).

Several situations are reached, depending on the spiro-compound: p53 is placed in the same position as the reference simulation, but its residues do not have the same intermolecular interactions (Figure 2A); p53 helix can be shifted with respect to the reference structure (Figure 2B); p53 helix twists laterally, partially losing its structure (Figure 2C).

**Figure 3:**
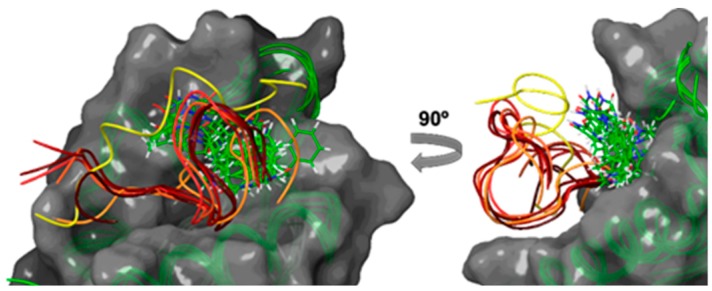
Snapshots from MD simulation of complex, ***exo-(3R,5R)-*****5a** bound to MDM2 and p53 at different simulation times (MDM2 in green, p53 from yellow to dark red): 0 ns, 100 ns, 200 ns, 300 ns, 400 ns, 600 ns, 800 ns, 1000 ns.

The only simulation where TAD1 is not able to fold back and remains completely unfolded is when ***exo*-*3R,5R*-5a** is present, as shown in Figure 3. This disordered structure is stable for more than 500 ns of simulation.

To our knowledge, there has never been comprehensively studied this system with molecular dynamic simulations so far, and our study suggests a correlation between the antiproliferative activity of a compound and the incapacity of p53 to fold back in presence of it. This correlation might be deeply studied and is beyond the aim of this exploratory work.

Intrigued by the results obtained with computational calculations and, in particular, with MD simulations, we considered it appropriate to investigate, in a preliminary way, the antiproliferative activity of our substrates against two cancer cell lines, theA549 human lung adenocarcinoma cells and human SH-SY5Y neuroblastoma cells. As already mentioned previously, these cancer cell lines were selected for the well-known pharmacological activity by spiro-oxindole derivatives, but not studied for spiro-indane substrates.

### 2.3. Antiproliferative Activity

As already mentioned above, different natural spirooxindoles like Spirotryprostatins A and B (Figure 1) showed relevant anticancer activities. Particularly, spirooxindole-pyrrolidines (Figure 1) revealed prominent cell inhibition against the A549 lung human adenocarcinoma cancer cell line at different concentrations (100, 50 and 25 μg/mL), exhibiting a best biological performance in spirooxindole-pyrrolidine derivatives alkylated on the *N*-oxindole ring with a long chain (i.e., hexyl) than the unsubstituted substrates, probably due to the increased lipophilicity [14].

In this regard, all synthetized compounds **5a**–**d** were assayed for in vitro cell growth control (%) against the A549 human lung cancer cell line using the most abundant diastereomeric form obtained for each compound (***exo*-5a**, ***exo*-5b**, ***exo*-5c**, ***exo*-5d**). Only for compound spiro (indane-isoxazolidine), *N*-Fluoro uracile **5c,** the *endo*-diastereomeric isomer was examined, hypothesizing that the compound **5c** an increased biological performance due to a probable bioisosteric effect of fluorine substituent, as detected for many generally active substrates [62,63]. Testing the two products ***exo*-5c** and ***endo*****-5c**, results for the cell growth percentage were comparable for both compounds. For this reason, we leaned towards the comparison only of *exo*-derivatives because of their major abundance.

**Figure 4:**
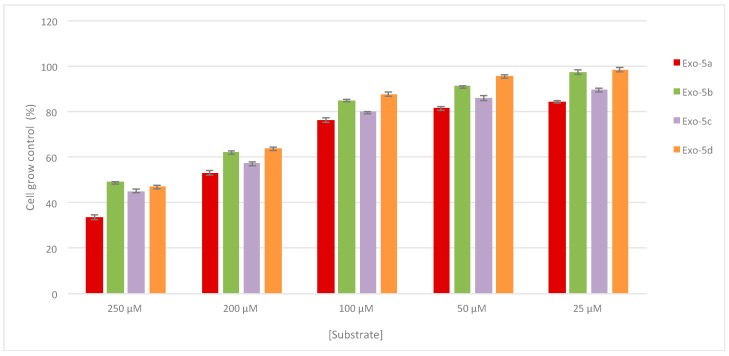
Antiproliferative effect of compounds ***exo*-5a-d** against A549 cells. The cellular-growth was measured by the SRB assay and reported as % relative to untreated control. Data are expressed as means ±SD of three independent observations (*p* < 0.05).

All compounds were checked for cell growth control at different concentrations (μM range), using SRB assay. In Figure 4, the cell growth control (%) in relation to [substrate] is reported for all compounds. The exact values along with standard deviation at the concentrations of 25, 50, 100, 200, 250 μM are reported in Table S1 of Supplementary Materials.

All substrates showed activity against the A549 cell line; however, some of the synthesized compounds exhibited a more evident inhibition performance. In fact, compound pyrimidine derivatives with C-5 substitution such as *N*-thymine derivative (***exo*-5a**) and *N*-(5-*F*-Uracil) derivative (***exo*-5c**) showed increased potencies than the C-5 unsubstituted analogue *N*-Uracil (***exo*-5b**). The purine nucleobase present on the *N*-adenine derivative ***exo*-5d** does not confer any improvement of antiproliferative capacity of substrate, which on the contrary, showed a slighter decrease of activity than the others.

**Figure 5:**
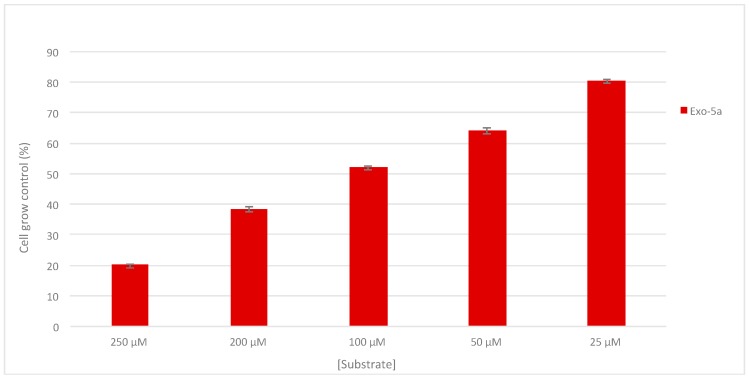
Antiproliferative effect of ***exo*-5a** against SH-SY5Y cells. The cell growth was measured by the SRB assay and reported as % relative to untreated control. Data are expressed as means ± SD of three independent observations (*p* < 0.05).

As pointed out by data in Figure 4 and highlighted also by the computational studies, compound ***exo*-5a** gave the most promising result and, therefore, it was chosen for additional studies on the human SH-SY5Y neuroblastoma cell line (Figure 5). The selection of this cell line was inducted by the fact that, to our knowledge, there are no spiro-isoxazolidine with indane core like ours tested on this cancer cell line. In this case, we observed a more encouraging antiproliferative performance than on the A549 cell line, manifesting a cell growth control of about 50% at a concentration of 50 μg/mL. The exact values along with standard deviation at the concentrations of 25, 50, 100, 200, 250 μM are reported in Table S2 of the Supplementary Materials.

The comparison of antiproliferative performance of the spirooxindole-pyrrolidines (Figure 1) reported in the literature [14] really shows similar results, prevalently for the more active substrate ***exo*-5a**. Anyway, it is good to specify in this contest that our intent is to consider these tests as proof of concept to better justify the results of the computational calculations, furnishing our contribution to understand the mechanisms of MDM2–p53 interaction disruption. So, our antiproliferative results may be considered as aforerunner for ulterior tests against cell lines more specific towards the inhibition of MDM2–p53 interactions by spiro-(indane isoxazolidine) derivatives.

## 3. Materials and Methods

### 3.1. General

Commercial starting materials were used without further purification. Reactions were monitored by TLC using silica plates 60-F264, commercially available from Merck (Milano, Italy). ^1^H and ^13^C-NMR spectra and two dimensional NMR spectra were recorded at 400 and 500 MHz and 100 and 125.7 MHz, respectively, in CDCl_3_ and DMSO-*d*_6_ using tetramethylsilane (TMS) as internal standard (Bruker Avance 500 MHz with a 5 mm TBO probe, Rheinstetten, Germany). Chemical shifts are given in parts per million and coupling constants in Hertz. The purity and the stereochemistry were established by NMR spectra (^1^H-NMR and NOESY experiments). Mass spectra were performed using a Thermo Scientific Q-Exactive^TM^ (Rodano, MI, Italy) mass spectrometer. Analysis was operated using electrospray with positive and negative polaritiy, at 140,000 resolving power, IT 500 ms, and ACG target = 3 × 10^6^, maximum injection time 200 ms by full scan analysis. Source conditions were: spray voltage 1.0 KV, capillary temperature: 250 °C; S-Lens RF Level: 50. The instrument was calibrated by Thermo calibration solutions prior to the beginning the analysis. The final substrates were further purified before biological assays with a semipreparative HPLC Waters 515 pump and injector, employing a Novapack silica column (19 × 30 mm, particle size 6 um) eluting with a isocratic mixture of hexane and isopropyl alcohol 95:5 and with a Waters dual wavelength detector 2487. MW-assisted reactions were performed in Synthos 3000 instrument from Anton Paar, equipped with a 4 × 24 MG5 rotor and an IR probe as external control of the temperature. 0.3–3 mL glass vials sealed with a dedicated PEEK screw-cup together with a reliable PTFE seal were used for all reactions.

For the synthesis of *N*-methyl-*C*-indanyl nitrone **3** see reference [42].

### 3.2. General Procedure for Synthesis of ***5a**–**d***

The nitrone (2 equiv.) and opportune *N*-nucleobase (1 equiv.) were grinded in a mortar, placed in apposite vessel and mixed in a vortex. The mixture was transferred to a microwave oven and was irradiated at 750 W, fixing T_max_ at 125 °C. After the appropriate time the crude oil was purified by flash chromatography with CHCl_3_/MeOH 9.75:0.25 *v*/*v*.

*Exo*-5′-Thyminyl-2′-methyl-spiro-[indane-3,3′-isoaxazolidine]. ***Exo*-5a** White solid, 77% yield. ^1^H-NMR (400 MHz, CDCl_3_): δ 1.76–1.90 (m, 1H, CH_2_), 1.94 (d, *J* = 1.17 Hz, 3H, CH_3_), 2.39 (s, 3H, CH_3_), 2.51–2.67 (m, 2H, CH_2_), 2.80–2.98 (m, 3H, CH_2_), 6.21 (dd, *J* = 5.61 Hz, 7.36 Hz, 1H, CH), 7.13–7.29 (m, 4H, Ar), 7.79 (s, 1H, 6-CH_Thy_), 9.39 (s_b_, 1H, NH_Thy_).^13^C-NMR (100 MHz, CDCl_3_): δ 12.85, 29.30, 30.23, 36.12, 51.69, 78.00, 82.29, 111.19, 123.64, 125.21, 126.99, 129.09, 135.93, 139.82, 144.40, 150.71, 164.21. ESI(+)-MS: *m*/*z* [M + H] calcd. for C_17_H_20_N_3_O_3_ 314.1499, found: 314.1497.

*Exo*-5′-Uracil-2′-methyl-spiro-[indane-3,3′-isoaxazolidine]. ***Exo*-5b** White solid, 76% yield. ^1^H-NMR (400 MHz, CDCl_3_): δ 1.72–1.91 (m, 1H, CH_2_), 2.39 (s, 3H, CH_3_), 2.53–2.66 (m, 2H, CH_2_), 2.80–2.94 (m, 2H, CH_2_), 2.94–3.02 (m, 1H, CH_2_), 5.78 (d, *J* = 8.08 Hz, 1H, 5-CH_Ura_), 6.19 (dd, *J* = 5.41 Hz, 7.31 Hz, 1H, CH), 7.14–7.30 (m, 4H, Ar), 8.03 (d, *J* = 8.08 Hz, 1H, 6-CH_Ura_), 9.54 (s_b_, 1H, NH_Ura_).^13^C-NMR (100 MHz, CDCl_3_): δ 29.44, 35.99, 51.94, 78.07, 82.68, 102.66, 123.69, 125.20, 127.05, 129.17, 139.59, 140.38, 144.34, 150.67, 163.71. ESI(+)-MS: *m*/*z* [M + H] calcd. for C_16_H_18_N_3_O_3_ 300.1343, found: 300.1337.

*Exo*-5′-(5-*F*-Uracil)-2′-methyl-spiro-[indane-3,3′-isoaxazolidine]. ***Exo*-5c** White solid, 89% yield. ^1^H-NMR (400 MHz, CDCl_3_): δ 1.78–1.90 (m, 1H, CH_2_), 2.40 (s, 3H, CH_3_), 2.52–2.64 (m, 2H, CH_2_), 2.80–2.94 (m, 2H, CH_2_), 2.95-3.04 (m, 1H, CH_2_), 6.17 (m, 1H, CH), 7.15–7.27 (m, 4H, Ar), 8.10 (d, *J* = 6.10 Hz, 1H, 6-CH_Ura_), 9.80 (s_b_, 1H, NH_Ura_).^13^C-NMR (100 MHz, CDCl_3_): δ 29.40, 30.18, 35.99, 51.92, 77.98, 82.93, 123.61, 124.63, 125.22, 127.10, 129.20, 139.67, 142.02, 144.32, 149.27, 157.16. ESI(+)-MS: m/z [M + H] calcd. for C_16_H_17_N_3_O_3_F 318.1248, found: 318.1240.

*Endo*-5′-(5-*F*-Uracil)-2′-methyl-spiro-[indane-3,3′-isoaxazolidine]. ***Endo*-5c** White solid, 89% yield. ^1^H-NMR (400 MHz, CDCl_3_): δ 2.12–2.22 (m, 1H, CH_2_), 2.24 (s, 3H, CH_3_), 2.29–2.44 (m, 1H, CH_2_), 2.55 (dd, *J* = 4.43 Hz, 14.02 Hz, 1H, CH_2_), 2.79–3.00 (m, 2H, CH_2_), 3.18 (dd, *J* = 7.75 Hz, 14.02 Hz, 1H, CH_2_), 6.26 (ddd, *J* = 1.37 Hz, 4.34 Hz, 7.75 Hz, 1H, CH), 7.16–7.28 (m, 4H, Ar), 8.05 (d, J = 6.17 Hz, 1H, 6-CH_Ura_), 8.57 (s_b_, 1H, NH_Ura_).^13^C-NMR (100 MHz, CDCl_3_): δ 29.72, 33.19, 37.61, 51.62, 77.89, 83.06, 124.71, 125.06, 126.91, 128.85, 139.41, 140.69, 141.75, 144.07, 148.75, 156.73. ESI(+)-MS: m/z [M + H] calcd. for C_16_H_17_N_3_O_3_F 318.1248, found: 318.1241.

*Exo*-5′-Adenyl-2′-methyl-spiro-[indane-3,3′-isoaxazolidine]. ***Exo*-5d** White solid, 86% yield. ^1^H-NMR (500 MHz, DMSO-*d*_6_): δ 1.82–2.02 (m, 1H, CH_2_), 2,45 (s, 3H, CH_3_), 2.64–2.81 (m, 1H, CH_2_), 2.82-3.03 (m, 2H, CH_2_), 3.04-3.19 (m, 1H, CH_2_), 6.44 (dd, *J* = 4.69 Hz, 7.72 Hz, 1H, CH), 7.88 (s_b_, 2H, NH_2_), 7.16–7.37 (m, 4H, Ar), 8.28 (s, 1H, CH_Ade_), 8.51 (s, 1H, CH_Ade_).^13^C-NMR (125 MHz, CDCl_3_): δ 33.78, 34.14, 41.87, 81.42, 82.03, 84.23, 123.11, 129.30, 131.04, 132.70, 133.10, 143.16, 144.92, 148.83, 153.98, 157.08, 160.43. ESI(+)-MS: m/z [M + H] calcd. for C_17_H_19_N_6_O 323.1615, found: 323.1623.

### 3.3. Computational Study

#### 3.3.1. Protein–LIGAND Docking Calculations

Conformational sampling of ligands **5a**–**d** and flexible docking with MDM2 were carried out using the Glide protocol in the Schrodinger modelling suite. The crystal structure of MDM2 in complex with a p53 (PDB entry 1YCR) was used as starting point for the receptor. Three different protein structures were considered, the first without p53, where p53 moiety was manually deleted, the second where the MDM2–p53 is conserved unaltered, and the third structure where p53 is manually moved 8 Å far from the groove. Then, the protein was prepared for docking calculations (hydrogen atoms added, water removed, hydrogen bonds optimized) using the Protein Preparation Wizard tool within the Maestro interface. For the grid calculations, a cubic box of 20 Å sides was centred on the hydrophobic cleft of MDM2. A constrained minimization of the receptor was carried out (OPLS3 force field, extended cutoffs of 8 Å for van der Waals interactions, 20 Å for electrostatic, and 4 Å for H-bonds, minimization with a limit of 5000 iterations and a convergence criterium of 0.01). Flexible docking in extra precision mode (XP) with sampling of ring conformations and nitrogen inversions was carried out. The energetically most favourable solution from the docking calculations was further minimized. The docking calculations were carried out with all four enantiomers of each of the five compounds.

#### 3.3.2. Molecular Dynamic Simulations

Protein and ligand structures were separately processed for Amber 16. Proteins were GAFF forcefield were used for ligands, while FF14SB was employed or proteins. Complexes were set in a explicit water TIP3P cubic box of 65 Å side, and charges were compensated. The system was minimized and heated to 300 K. MD simulations were performed in the isothermal-isobaric ensemble (NPT) at 300 K and 1 bar, under the Periodic Boundary Conditions, using the Langevin thermostat, Berendsen barostat, non-bonded interaction cutoff set to 10 Å, SHAKE approximation for hydrogen atoms with a time step of 2 fs. The trajectory was recorded at 10 ps intervals for at least 500 ns. Analysis of the trajectories was carried out using tools of AMBER and VMD [64].

### 3.4. Biological Evaluation

#### 3.4.1. Cell Culture

A549 cells were purchased from ATCC and were maintained at 37 °C and in a 5% CO_2_ environment in complete media constituting basal medium Roswell Park Memorial Institute (RPMI)-1640 supplemented with 10% fetal bovine serum (FBS), 1% penicillin streptomycin and 1% L-glutamine. Briefly, cells in the log phase of growth were harvested and seeded at a density of 1.25 × 10^5^ cells in 96-well plates and cultured till 80% confluency for 24 h at 37 °C, 5% CO_2_. The media was then replaced with 200 µL of different serially diluted compounds in complete media (with a final concentration of 0.2% DMSO) to obtain a concentration range of 250–25 µM (n = 3) with 10% dimethyl sulfoxide (DMSO) serving as a positive control and 0.2% DMSO in complete media serving as negative control. The plates were further incubated for 48 h. The human SH-SY5Y neuroblastoma cell line, used for compound ***Exo*-5a**, was purchased from ATCC, the Global Bioresource Center, and maintained in Dulbecco’s modified Eagle’s medium (DMEM) (Sigma-Aldrich Ltd.) supplemented with 5 mg mL^−1^ penicillin/streptomycin, 10 mM l-glutamine, and 10% (*v*/*v*) fetal bovine serum (FBS). Cells were kept in an incubator at 37 °C with a 5% CO_2_ atmosphere.

#### 3.4.2. Sulforhodamine B assay

The human lung adenocarcinoma cell line A549 (passage number 16–18) was used to study the in vitro cell growth control (%) assay of the spyrocyclic compounds (***exo*-5a**, ***exo*-5b**, ***exo*-5c**, ***endo*-5c**, ***exo*-5d**) at different concentration (250 μM, 200 μM, 100 μM, 50 μM, 25 μM) and hence validate its antiproliferative activity. The neuroblastoma cell line SH-SY5Y was used only to test the in vitro cell growth control (%) assay of spirocyclic compound ***exo*-5a** at different concentration (250 μM, 200 μM, 100 μM, 50 μM, 25 μM). After two passages, cells were plated at a density of 2 × 10^4^ per well in a 96-well microplate for the SRB assay. For the Sulforhodamine B (SRB) assay at the end of the incubation period, cell fixation was carried out by addition of 50 µL of 40% trichloroacetic acid to each well supernatant for 1 h at 4 °C. The plates were then washed with deionized water four times and were allowed to air-dry at 25 °C overnight. Cell staining was carried out by addition of 100 µL of 0.057% of SRB solution and left at room temperature for 1 h. The plates were washed with 1% acetic acid solution to remove the unstained SRB reagent four times and allowed to air-dry. 200 µL of 10 mM Tris base (pH 10.5) was added to solubilize the protein bound SRB dye and optical density (OD) was measured by spectroscopy (SpectraMAX Plus 384, Molecular Devices, Berkshire, UK) at 510 nm after shaking the plates mechanically for 5 min. The plate-by-plate examination of the test wells relative to control wells was employed to determine percent growth, and the ratio of the average absorbance in the test well to the average absorbance in the control wells × 100 was determined.

#### 3.4.3. Statistical Analysis

The results are expressed by mean ± S.E.M. from at least three independent experiments. For statistical comparisons, quantitative data were analysed by one-way analysis of variance (ANOVA) followed by Tukey-test according to the statistical program SigmaStat1 (Jandel Scientific, Chicago, IL, USA). A *p*-value less than 0.05 was regarded as significant.

## 4. Conclusions

In conclusion, we have synthesized regio- and diastereomerically pure nucleobase-containing spiro (indane-isoxazolidine) derivatives by MW-assisted 1,3 dipolar cycloaddition between indanyl nitrones and *N*-vinylnucleobases in high yields and very short reaction times with respect to classical cycloaddition conditions. We carried out computational calculations such as docking and molecular dynamics to study the hypothetical inhibition of MDM2–p53 interaction by our substrates. These studies highlighted particular comportments, prevalently of the molecule ***exo*-5a**, that revealed to be the most active in the biological tests carried out against A549 human lung adenocarcinoma cells and SH-SY5Y neuroblastoma cells. We are aware that these studies are preliminary and that, in future, specific cell lines to confirm inhibition of MDM2–p53 interaction should probably be done, but we believe that these first results are really encouraging to demonstrate, inter alia, that the indane core might be a potential substitute of the oxindole ring, which is so far, the most frequently used in the inhibition of MDM2–p53 interaction.

## Figures and Tables

**Table 1:** Reaction conditions and results of s*olvent-free* synthesis of cycloadducts **5a**–**d**.

Entry ^a^	Time (min)	Product	Yield (%)	*Exo*/*endo* Ratio
1	40	**5a**	77	75:25
2	40	**5b**	76	78:22
3	45	**5c**	89	77:23
4	45	**5d**	86	75:25

^a^ MW = 750 W; T = 125 °C.

## Supplementary Materials

The following are available online. Characterization: NMR and HRMS spectra of **5a**–**5d** compounds. Biological assay. Table S1 and Table S2: Control of cell growth of compounds **5a**–**d** against A549 and SH-SY5Y cells, respectively. Docking studies. Table S3: Docking score of input ligands to MDM2; Figure S1. Stacked plot of minimum energy docking poses for **5a**–**d** derivatives (A). Detail of ***exo-(3R,5R)*-5a** in green superimposed to p53 in red and grey (B). Figure S2. Snapshot from MD simulation of ***exo-(3R,5R)*-5a** bounded to MDM2 (A). Snapshots from MD at different simulation times of ***endo-(3R,5S)-*****5a** bounded to complex MDM2–p53 (B). Figure S3. Snapshots from MD simulation of unbounded p53 and MDM2 at different simulation times. MDM2 in green, p53: starting structure (yellow), 100 ns (light orange), 200 ns (orange), 300 ns (light red), 400 ns (red), 500 ns (dark red).
