# Supplementary material for: Synthesis, Biological and In Silico Evaluation of Pure Nucleobase-Containing Spiro (Indane-Isoxazolidine) Derivatives as Potential Inhibitors of MDM2–p53 Interaction
Source: Molecules. 2019 Aug 10;24(16):2909. doi: 10.3390/molecules24162909 (PMC6719244; doi:10.3390/molecules24162909)
Supplement: Supplementary file 1 [file molecules-24-02909-s001.pdf]

## Supporting Information

# Synthesis, Biological and *in Silico* Evaluation of Pure Nucleobase-Containing Spiro (Indane-Isoxazolidine) Derivatives as Potential Inhibitors of MDM2-p53 Interaction


*Loredana Maiuolo,*[a] Vincenzo Algieri,[a] Beatrice Russo,[a] Matteo Antonio Tallarida,[a] Monica Nardi,[b] Maria Luisa Di Gioia,[c] Zahra Merchant,[d] Pedro Merino,[e] Ignacio Delso,*[f] Antonio De Nino*[a]*

[a] Dipartimento di Chimica e Tecnologie Chimiche, Via P. Bucci, cubo 12C, Università della Calabria, 87036 Rende (CS), Italy;

[b] Dipartimento di Scienze della Salute, Università Magna Græcia, Viale Europa, Germaneto, Catanzaro, Italy

[c] Dipartimento di Farmacia e Scienze della Salute e della Nutrizione, Edificio Polifunzionale, Università della Calabria, 87036 Rende (CS), Italy

[d] UCL School of Pharmacy, Department of Pharmaceutics, London, United Kingdom.

[e] Unidad de Glicobiología, Instituto de Biocomputación y Física de Sistemas Complejos (BIFI), Universidad de Zaragoza, 50009 Zaragoza, Spain

[f] Departamento de Síntesis y Estructura de Biomoléculas, Instituto de Síntesis Química y Catálisis Homogénea (ISQCH), Universidad de Zaragoza-CSIC, 50009 Zaragoza, Spain.



**NMR and ESI(+)-MS spectra……….……………………………………….…...S-2**

**Biological Assays……………………………………………………………S-21**

**Computational Studies……………………………………………………..S-22**

# *Exo*-5’-Thyminyl-2’-methyl-spiro-[indane-3,3’-isoaxazolidine]. (*Exo*-5a)

## $^1$H NMR

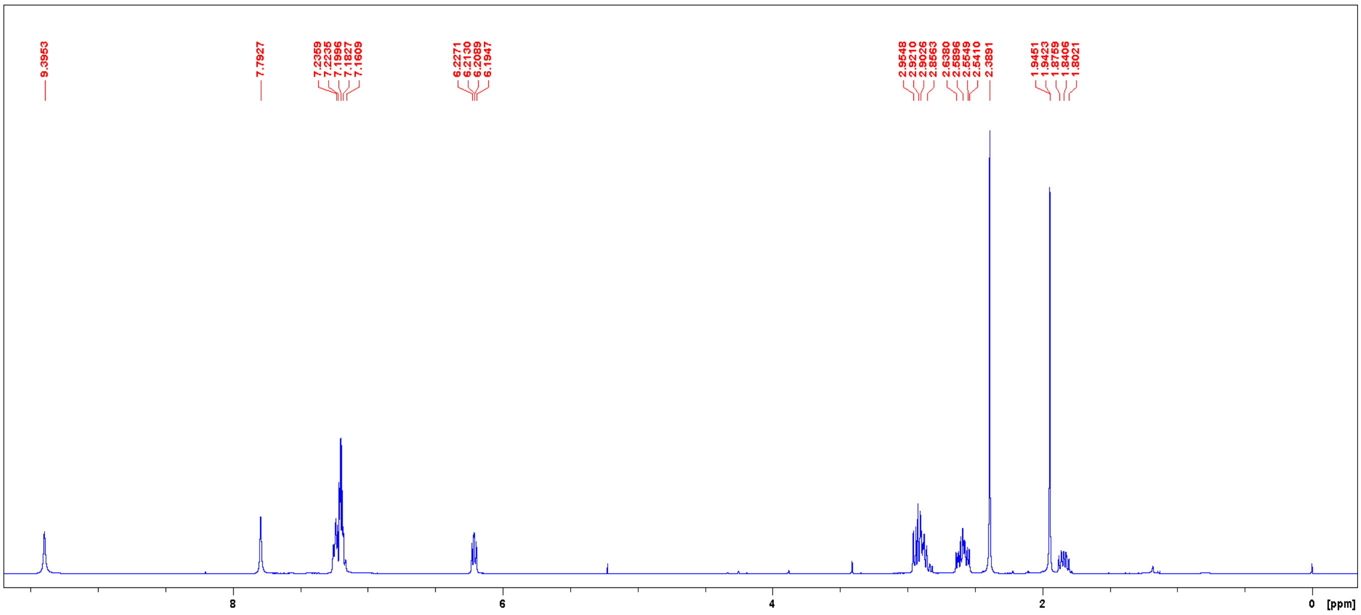


## APT NMR

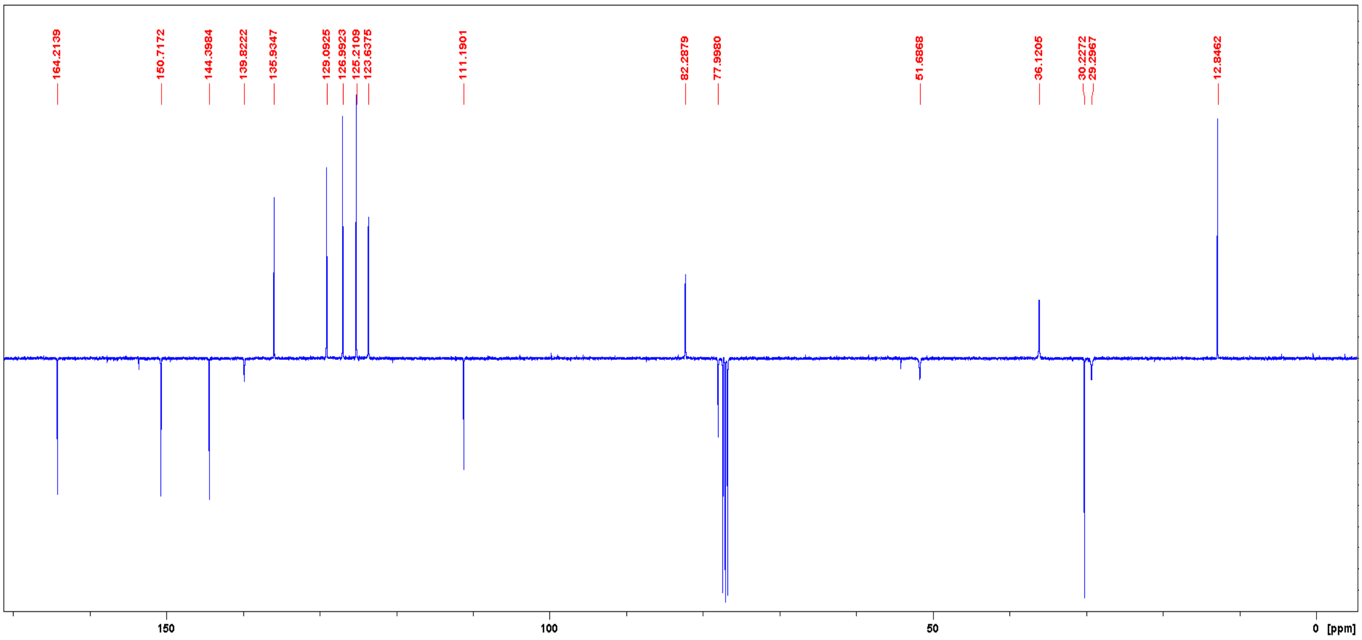

**COSY-NMR**

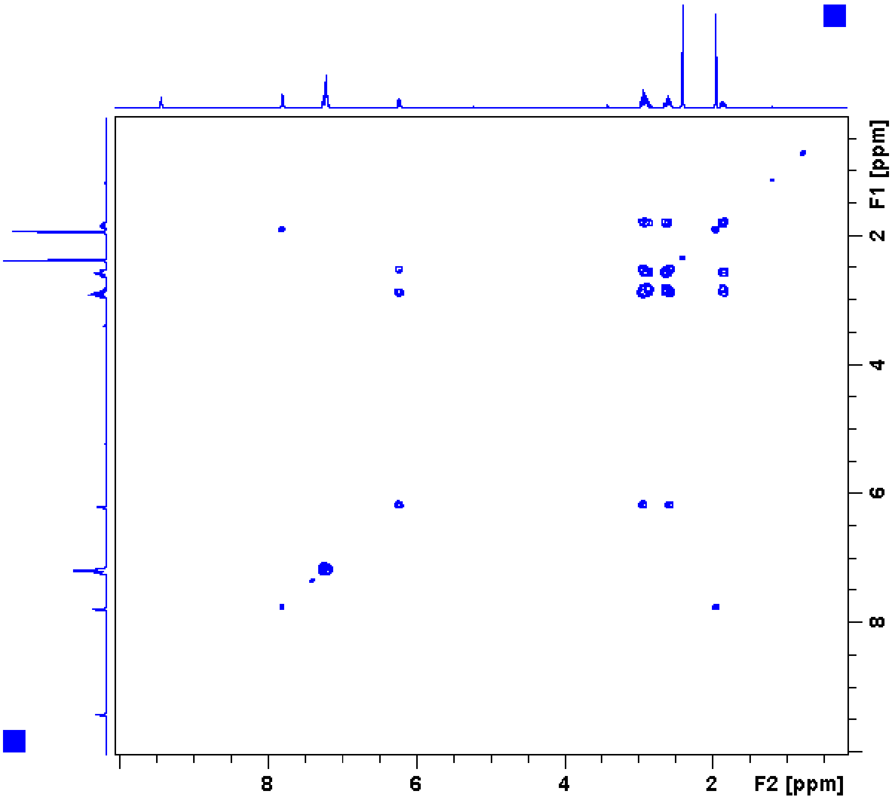


**HSQC-NMR**

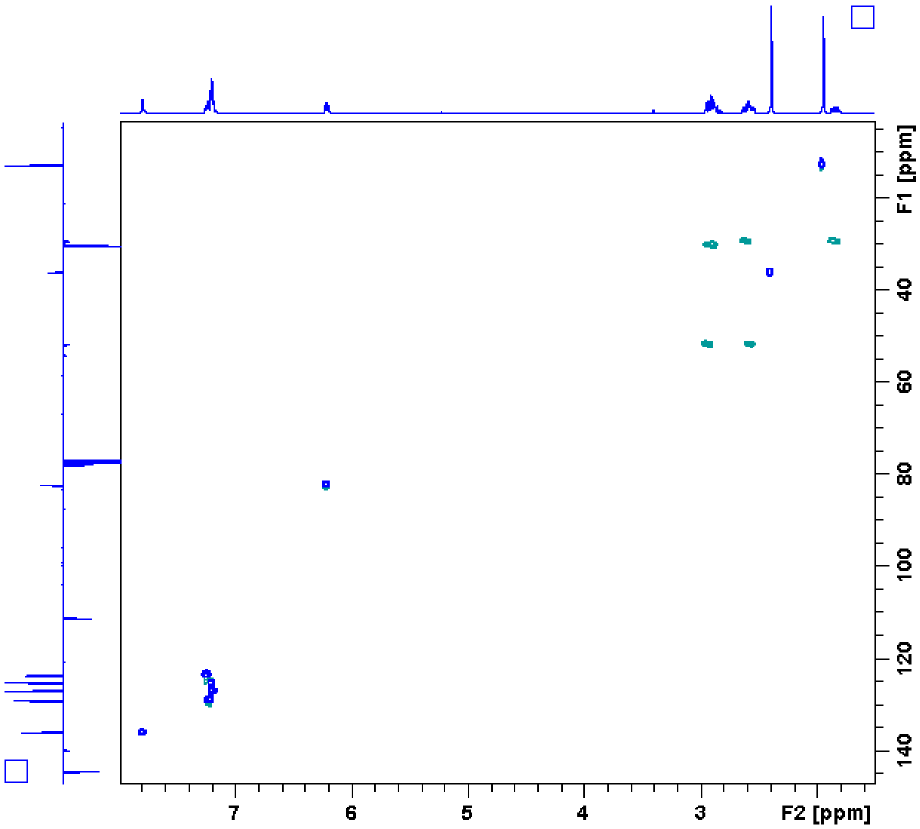

## HMBC-NMR

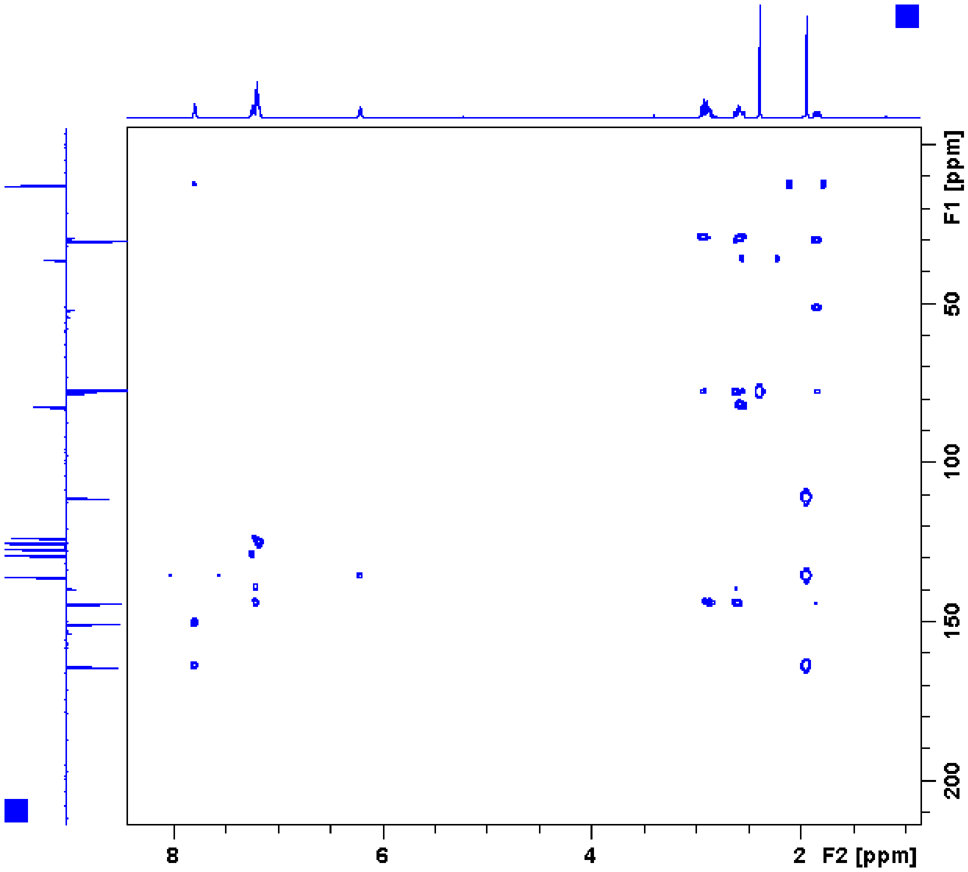


## NOESY-NMR

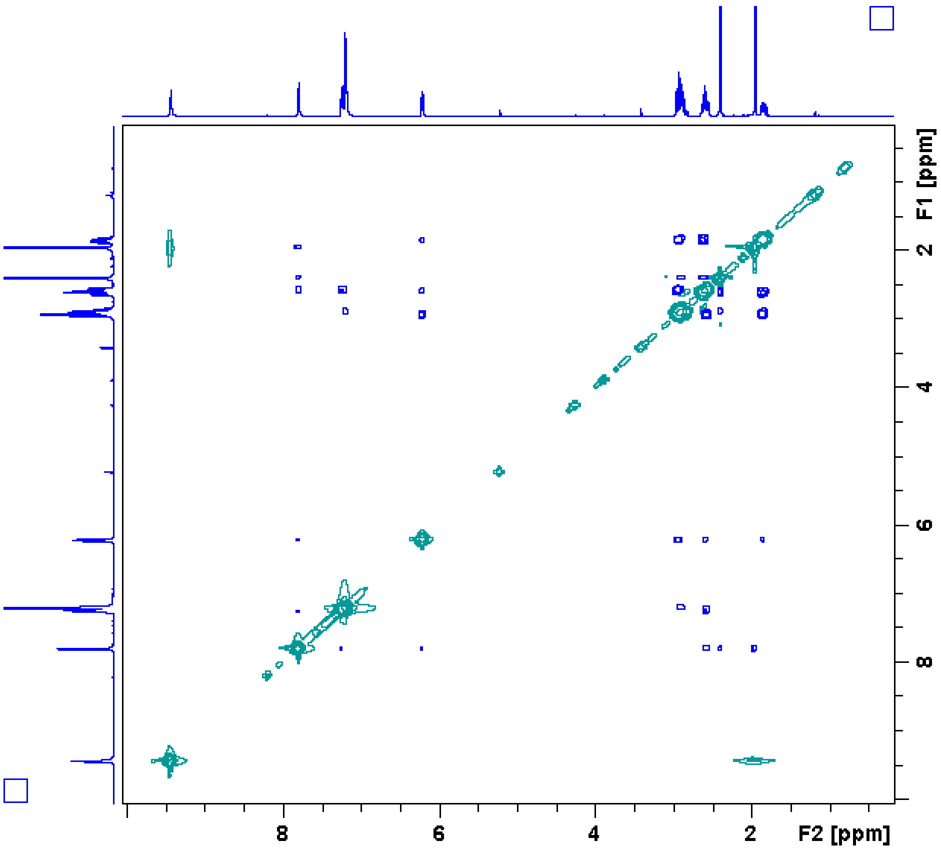

## ESI(+)-MS

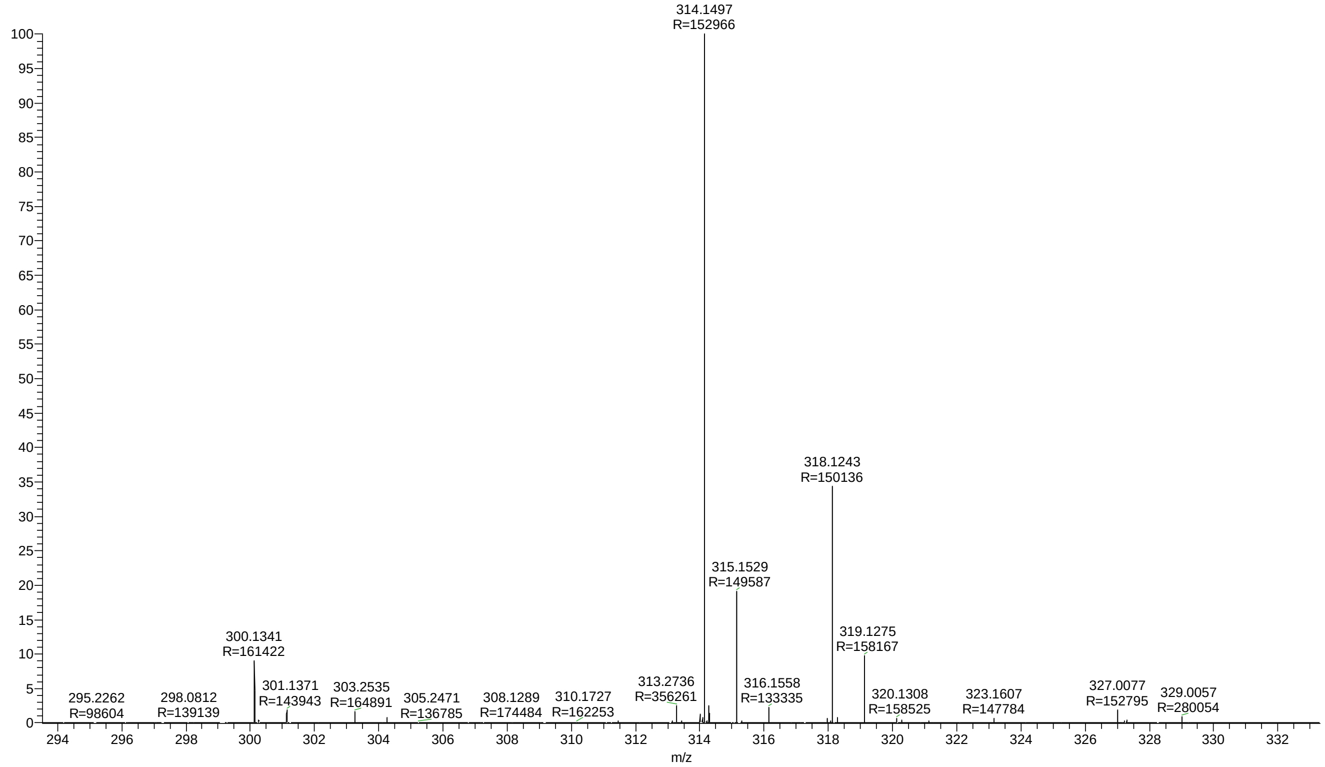
314.1497
R=152966
318.1243
R=150136
315.1529
R=149587
319.1275
R=158167
300.1341
R=161422
301.1371
R=143943
303.2535
R=164891
305.2471
R=136785
308.1289
R=174484
310.1727
R=162253
313.2736
R=356261
316.1558
R=133335
320.1308
R=158525
323.1607
R=147784
327.0077
R=152795
329.0057
R=280054
295.2262
R=98604
298.0812
R=139139
m/z

## *Exo*-5'-Uracil-2'-methyl-spiro-[indane-3,3'-isoaxazolidine]. (*Exo*-5b)

### $^{1}$H NMR

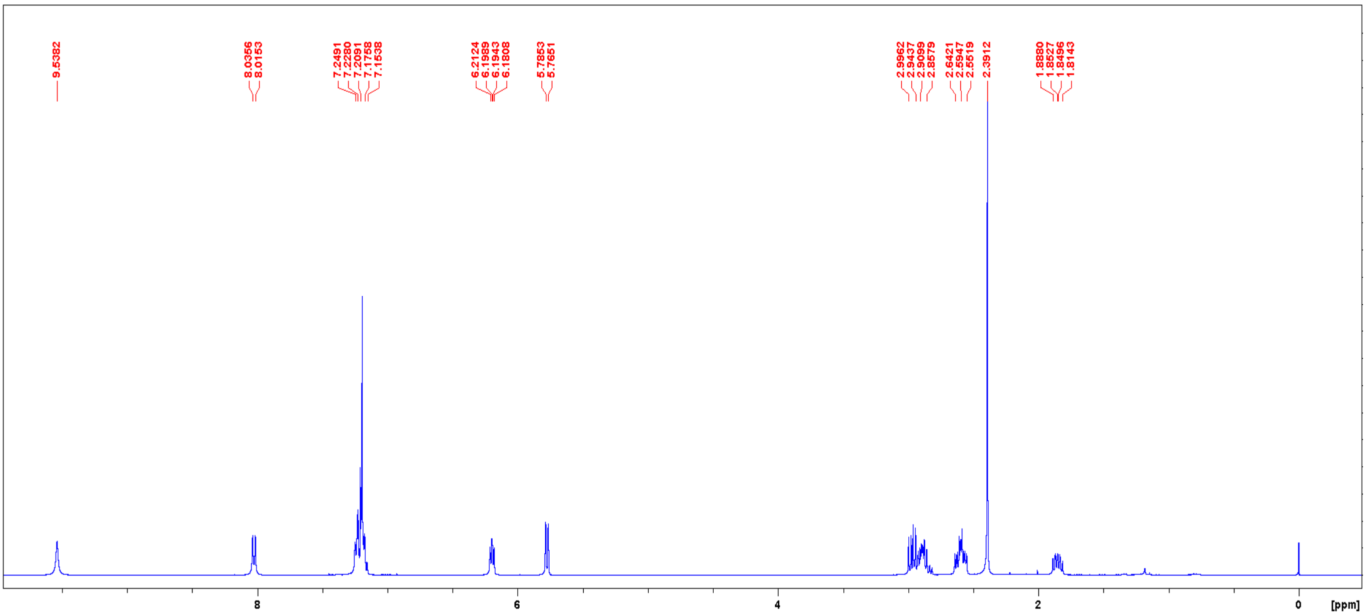


### APT NMR

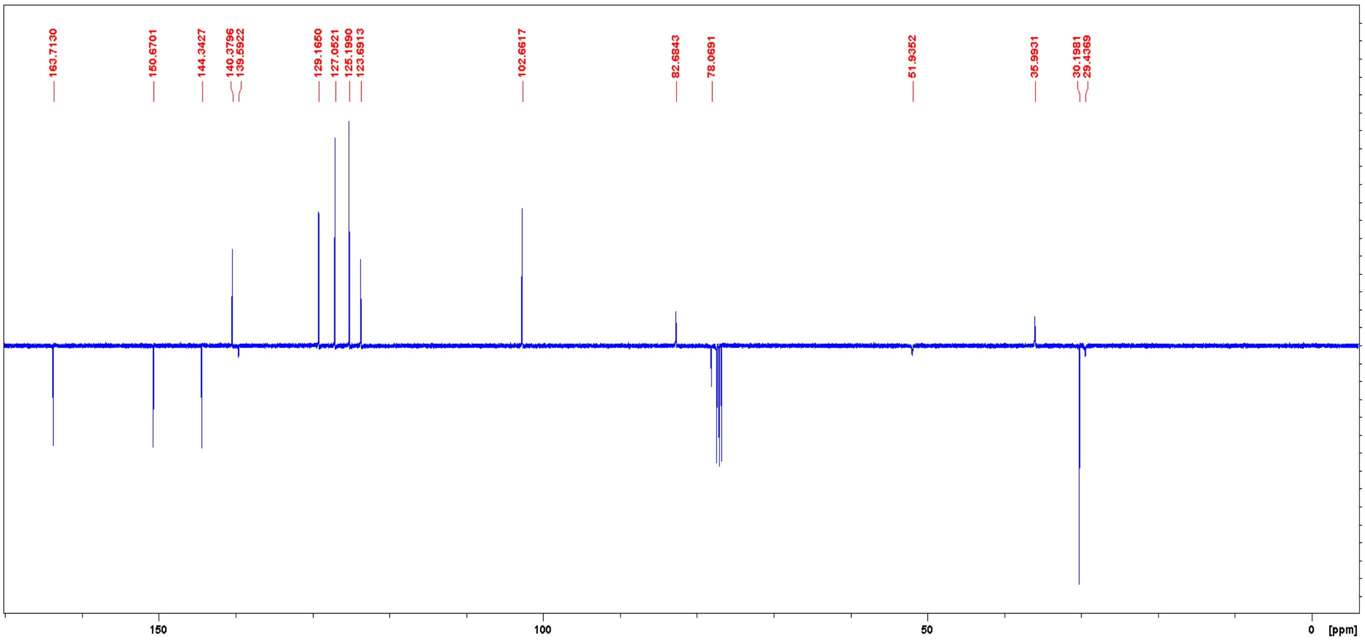

## COSY NMR

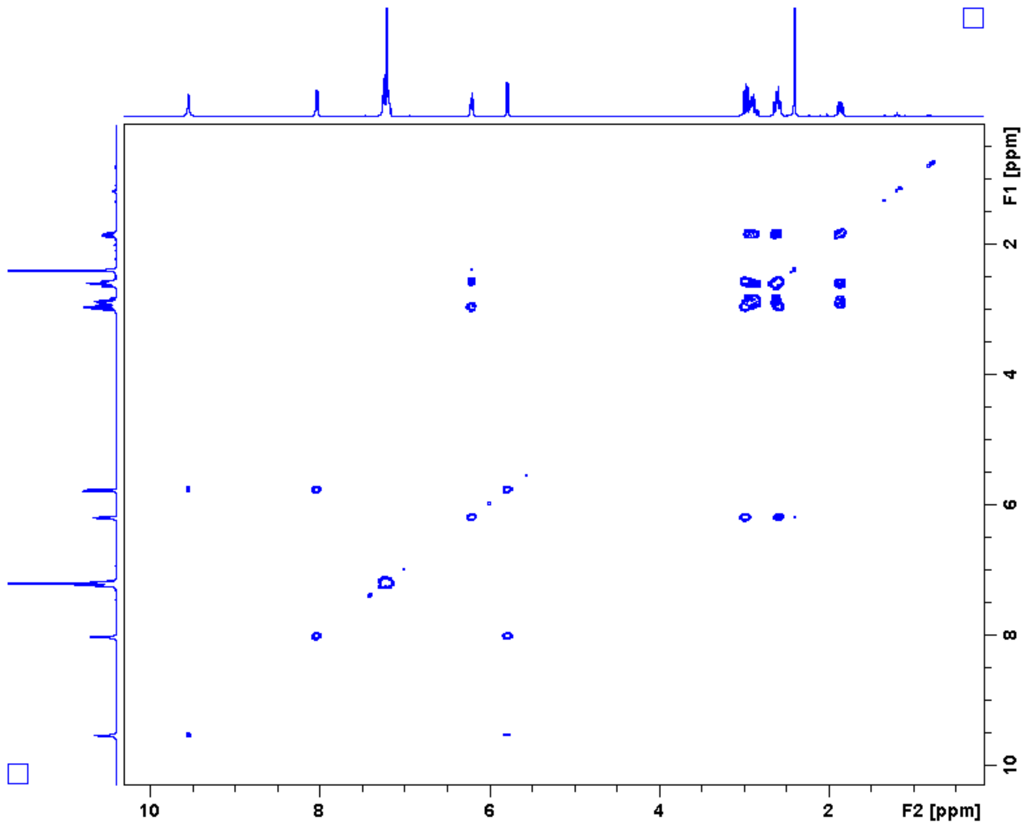


## HSQC NMR

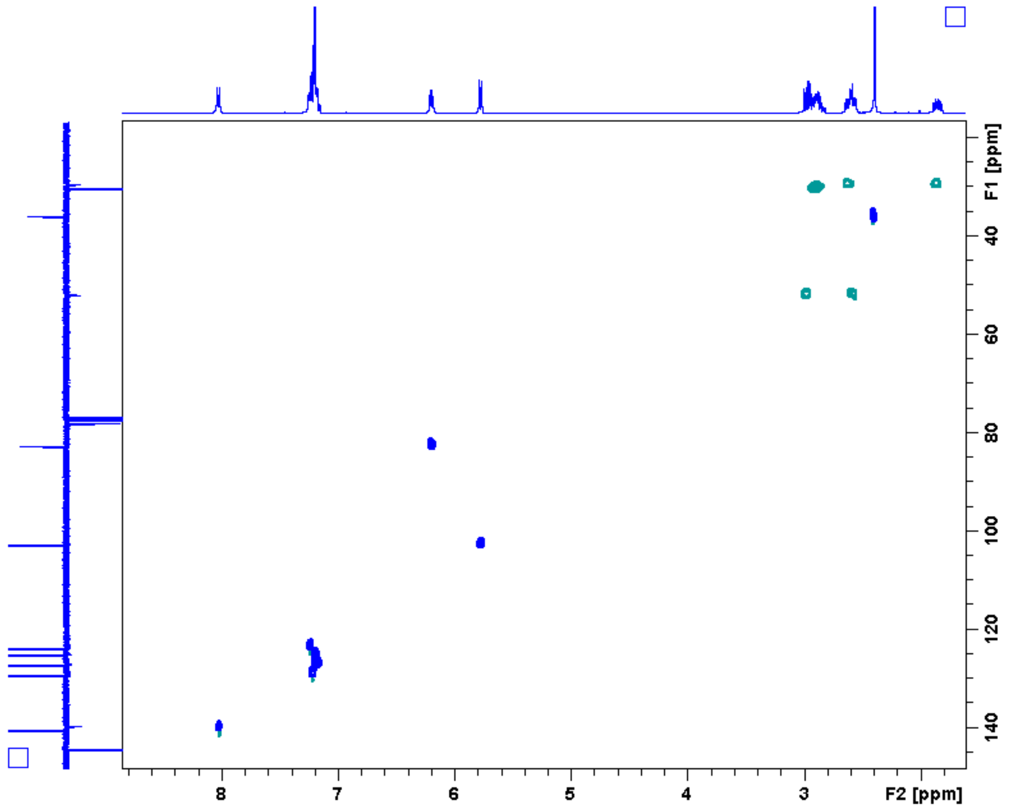

HMBC NMR

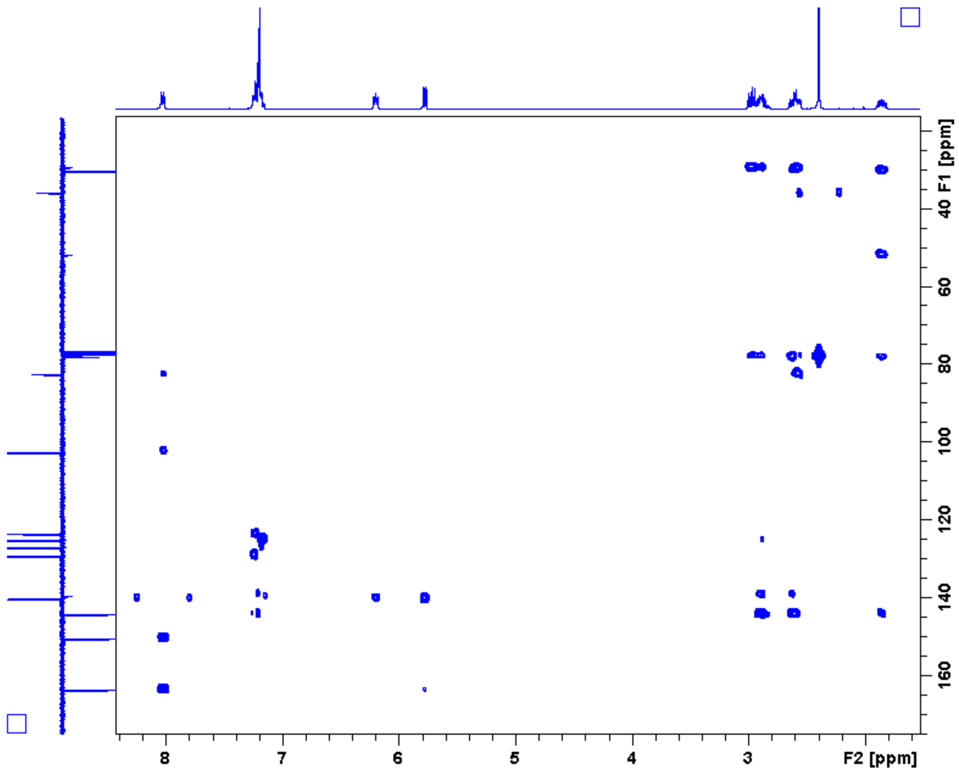


NOESY NMR

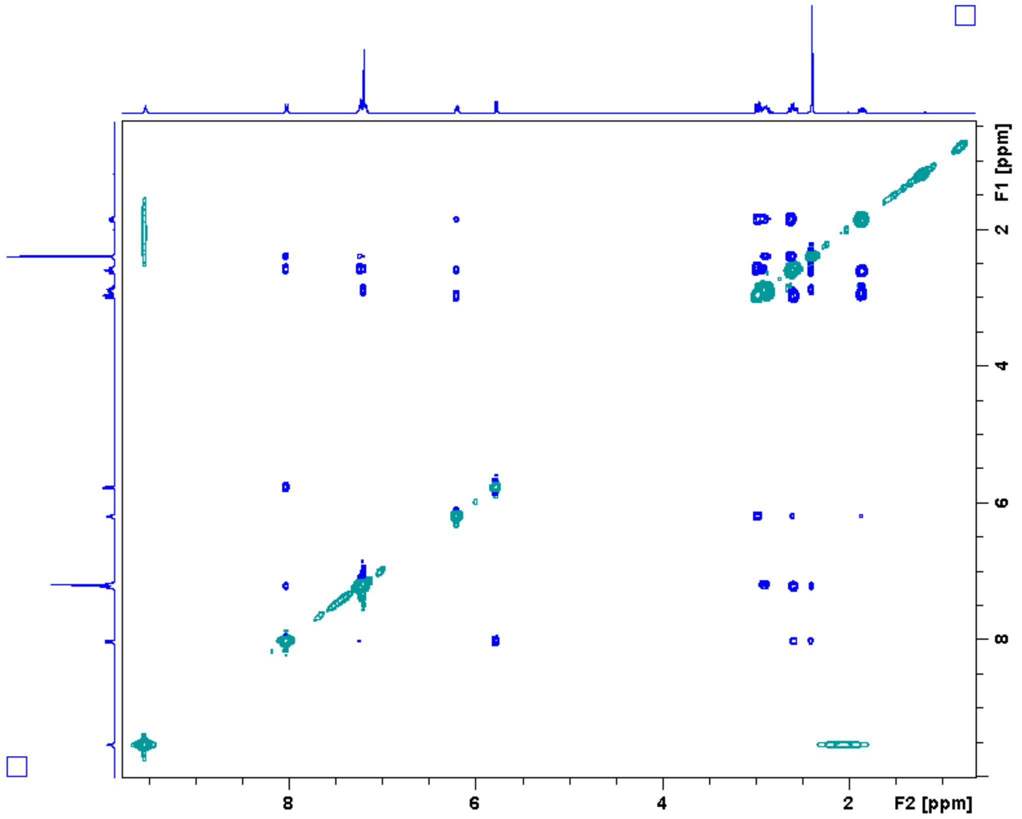

**ESI(+)-MS**

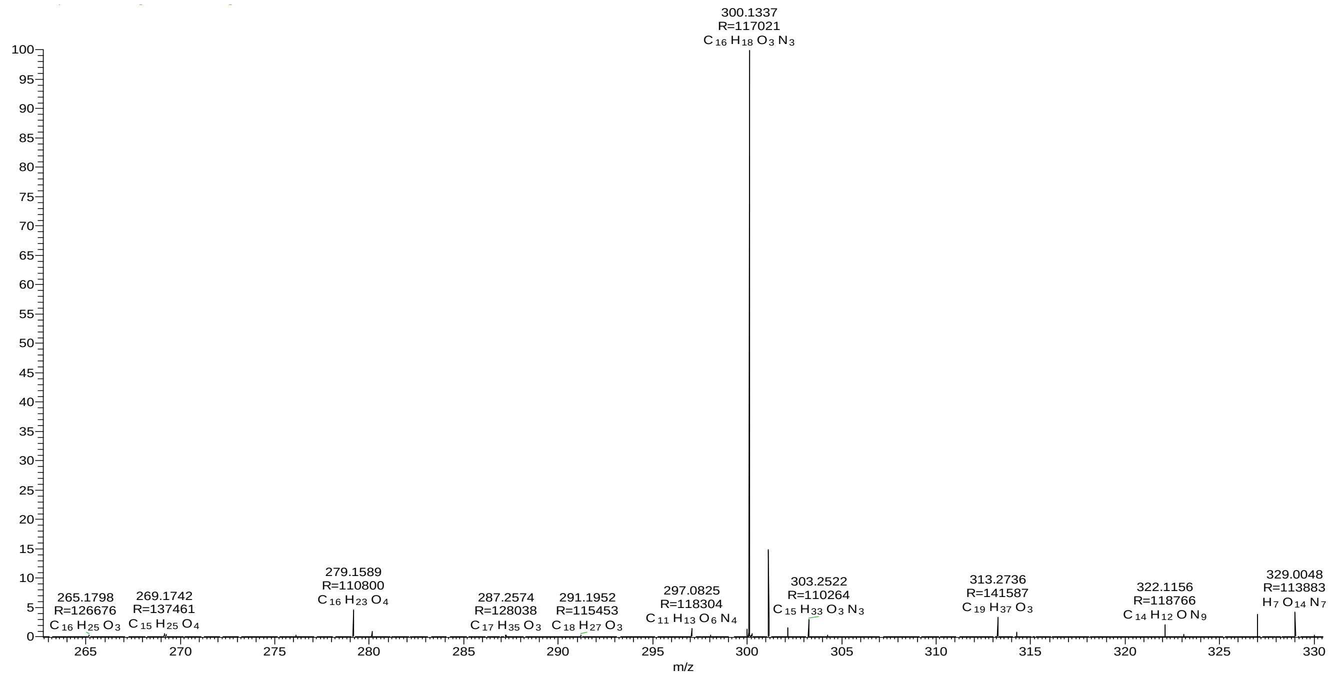
300.1337
R=117021
C16 H18 O3 N3
265.1798
R=126676
C16 H25 O3
269.1742
R=137461
C15 H25 O4
279.1589
R=110800
C16 H23 O4
287.2574
R=128038
C17 H35 O3
291.1952
R=115453
C18 H27 O3
297.0825
R=118304
C11 H13 O6 N4
303.2522
R=110264
C15 H33 O3 N3
313.2736
R=141587
C19 H37 O3
322.1156
R=118766
C14 H12 O N9
329.0048
R=113883
H7 O14 N7
m/z

## *Exo*-5'-(5-F-Uracil)-2'-methyl-spiro-[indane-3,3'-isoaxazolidine]. (*Exo*-5c)

### $^{1}$H NMR

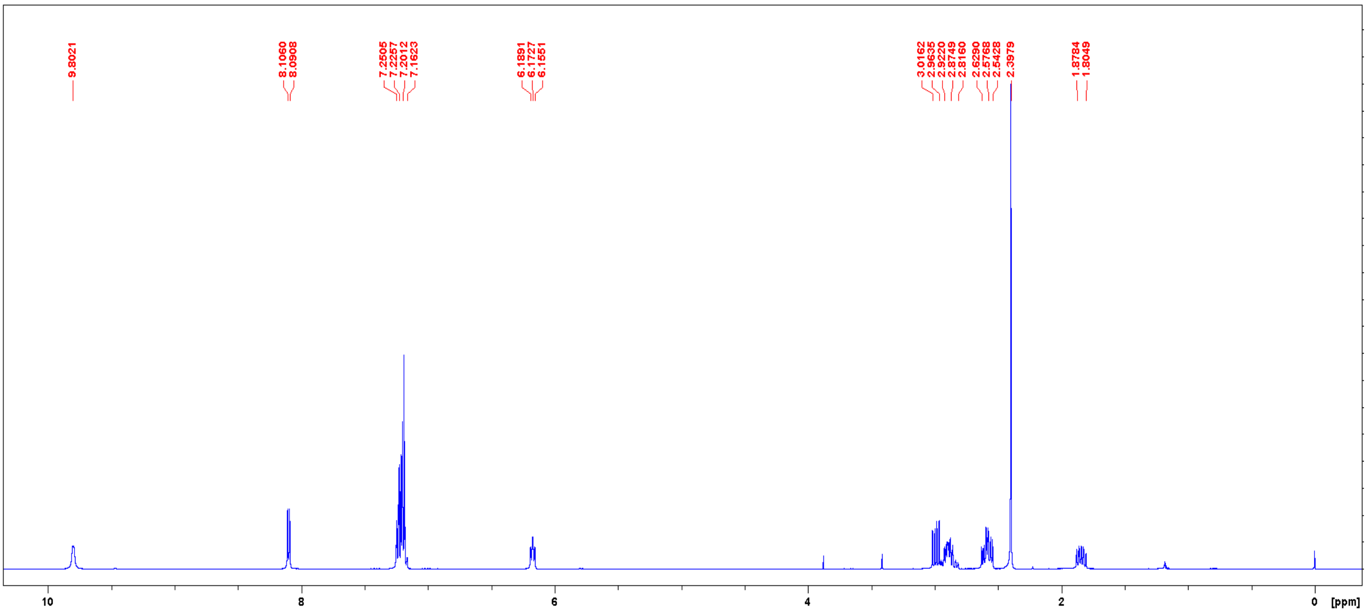


### APT NMR

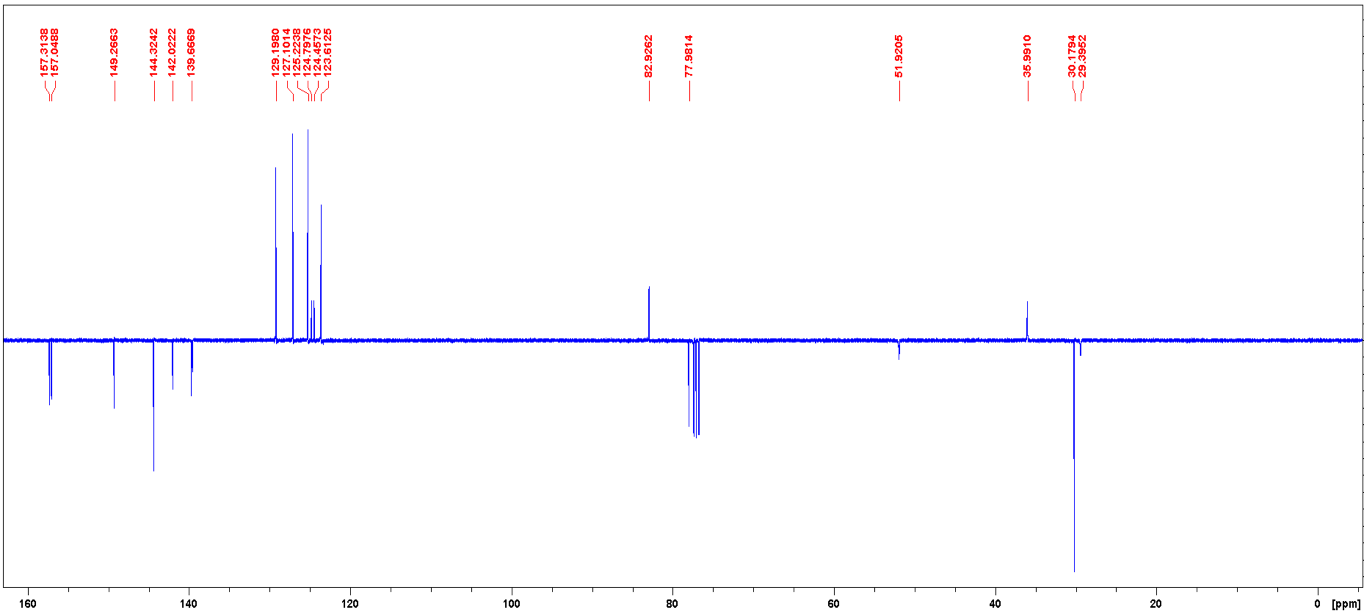

**COSY NMR**

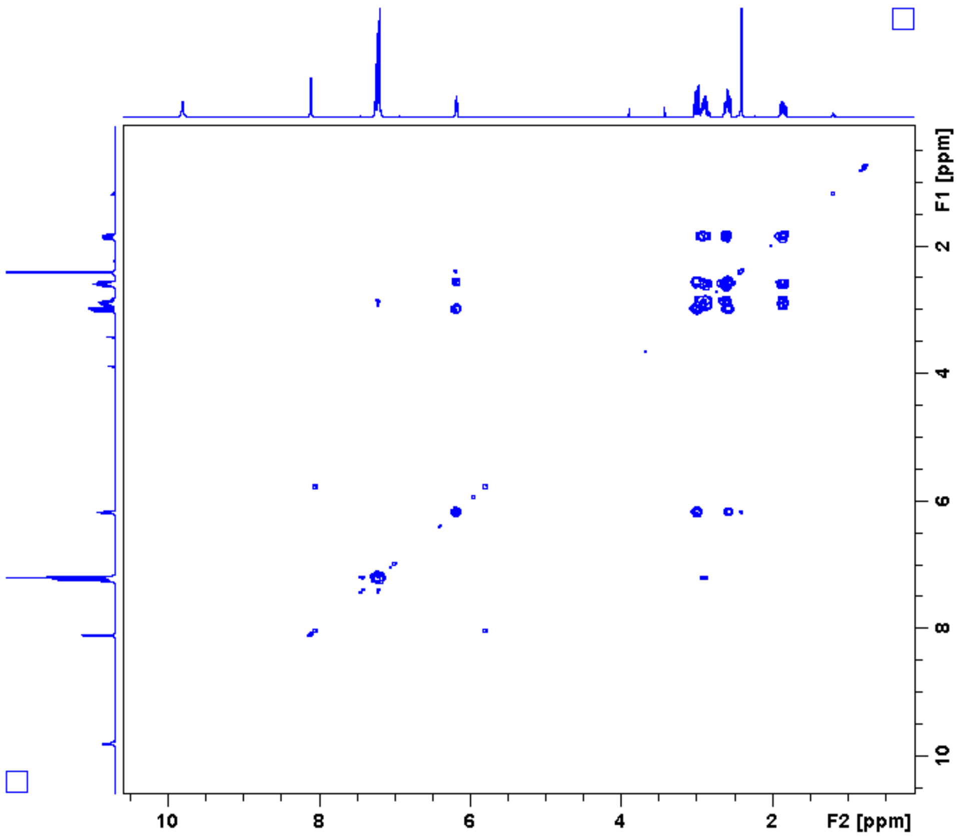


**HSQC NMR**

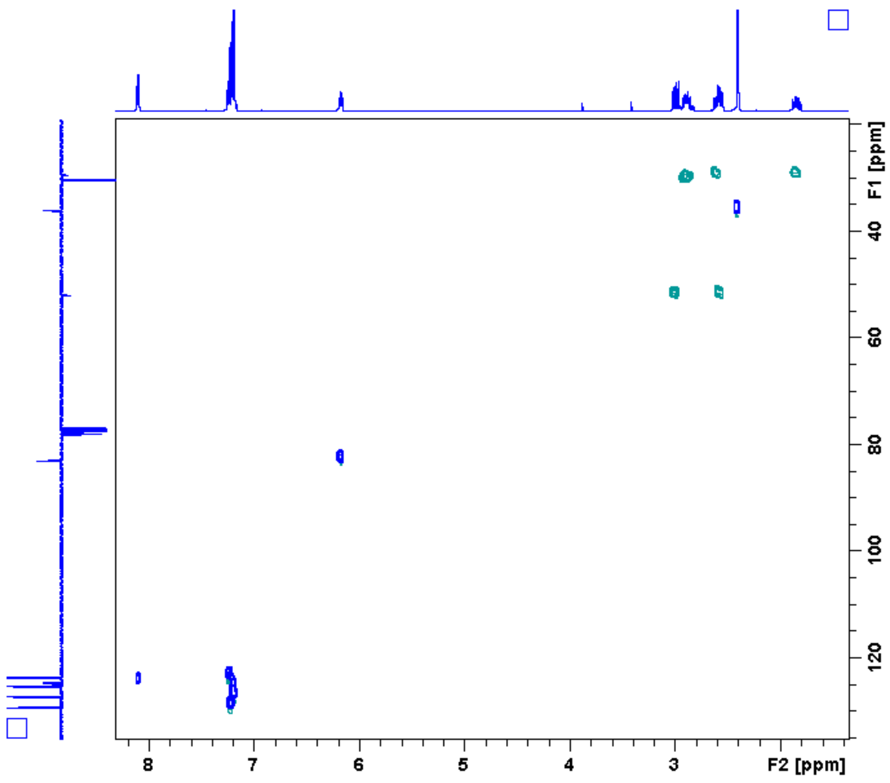

HMBC NMR

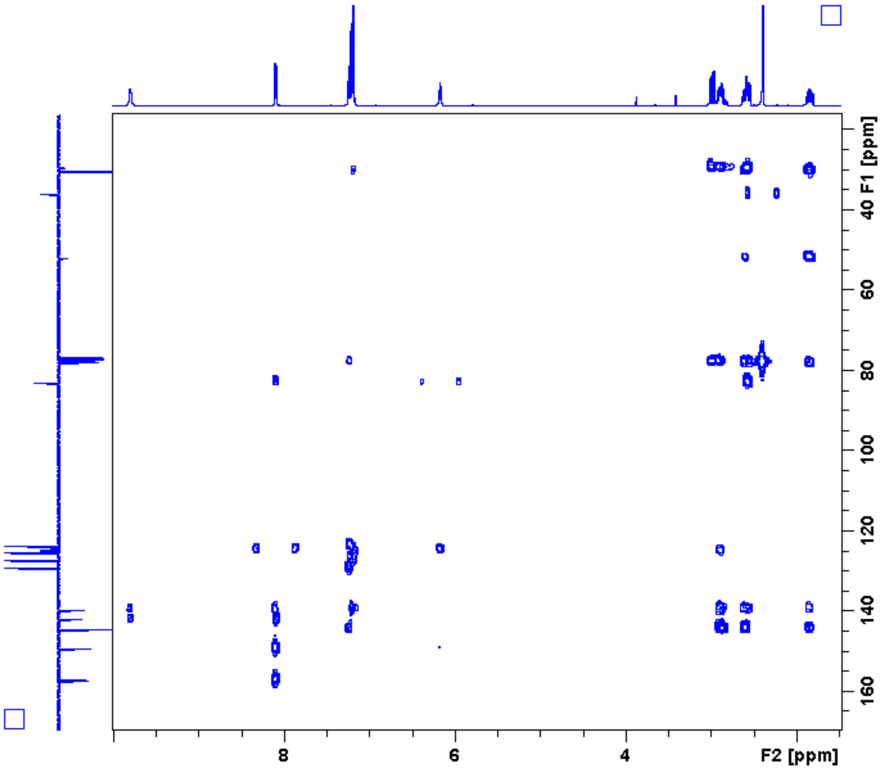


NOESY NMR

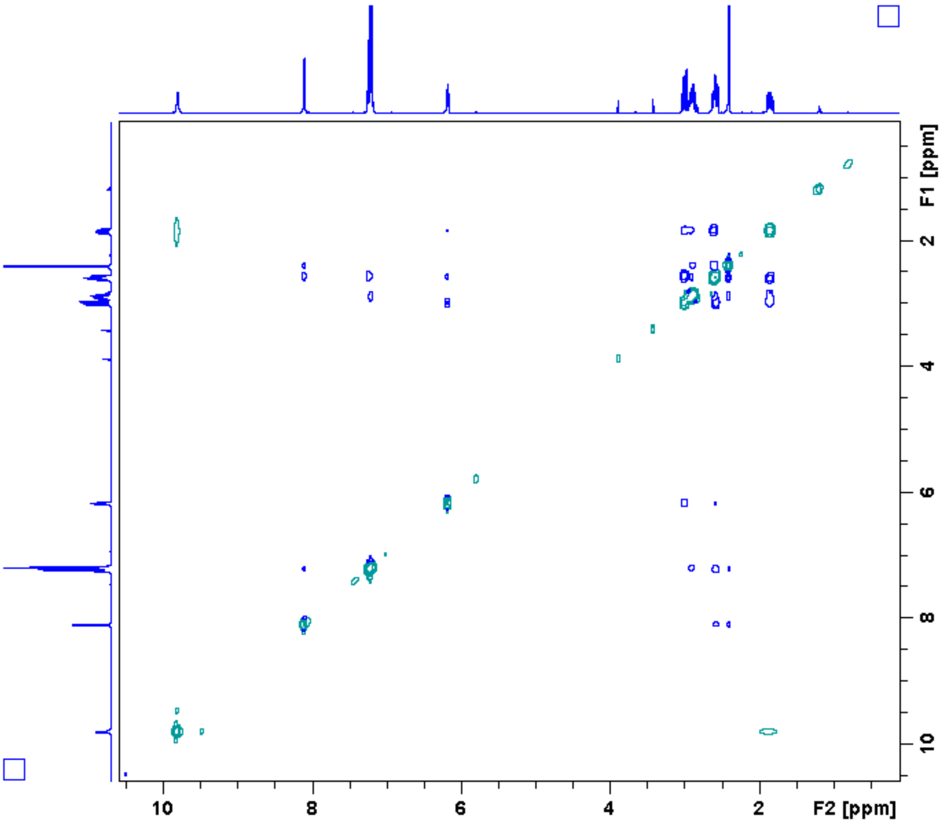

## ESI(+)-MS

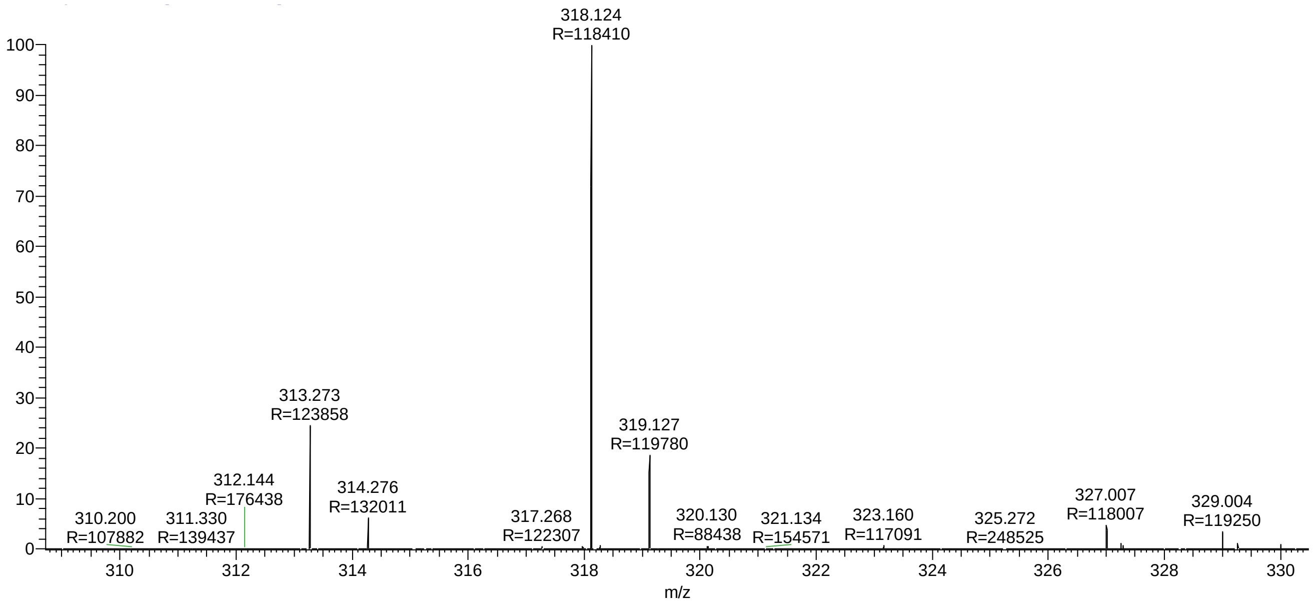

318.124
R=118410
313.273
R=123858
319.127
R=119780
312.144
R=176438
314.276
R=132011
310.200
R=107882
311.330
R=139437
317.268
R=122307
320.130
R=88438
321.134
R=154571
323.160
R=117091
325.272
R=248525
327.007
R=118007
329.004
R=119250
m/z

*Endo*-5'-(5-F-Uracil)-2'-methyl-spiro-[indane-3,3'-isoaxazolidine]. (*Endo*-5c)

## $^{1}$H NMR

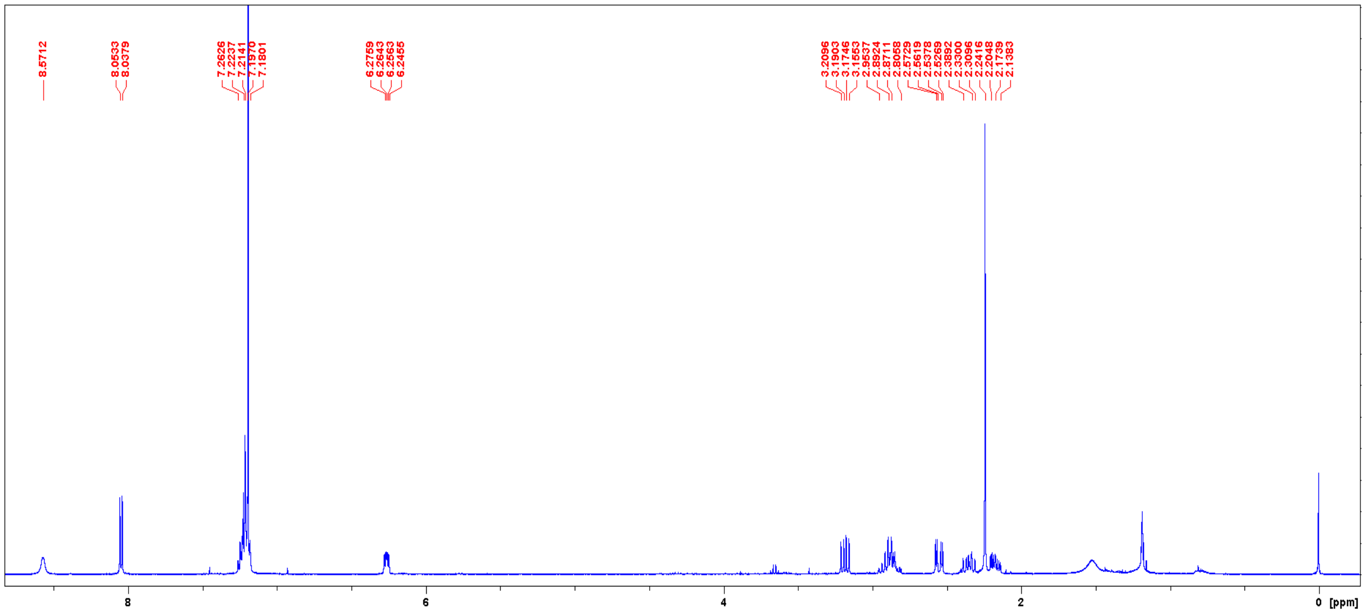

## APT NMR

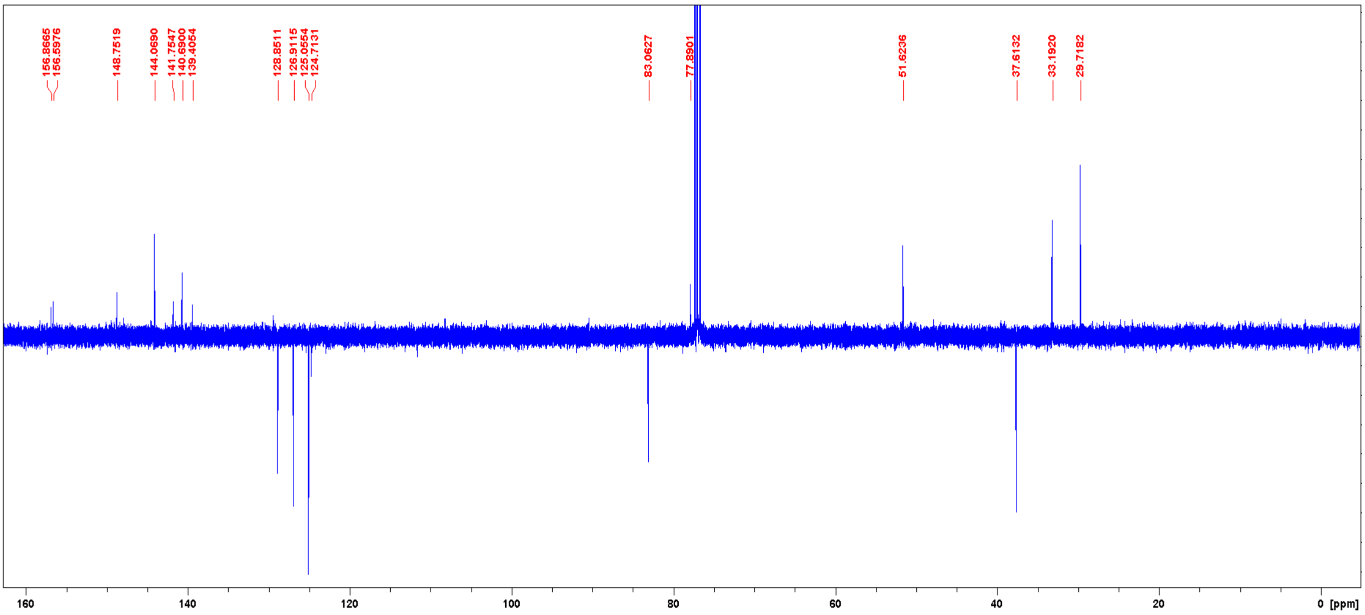

## COSY NMR

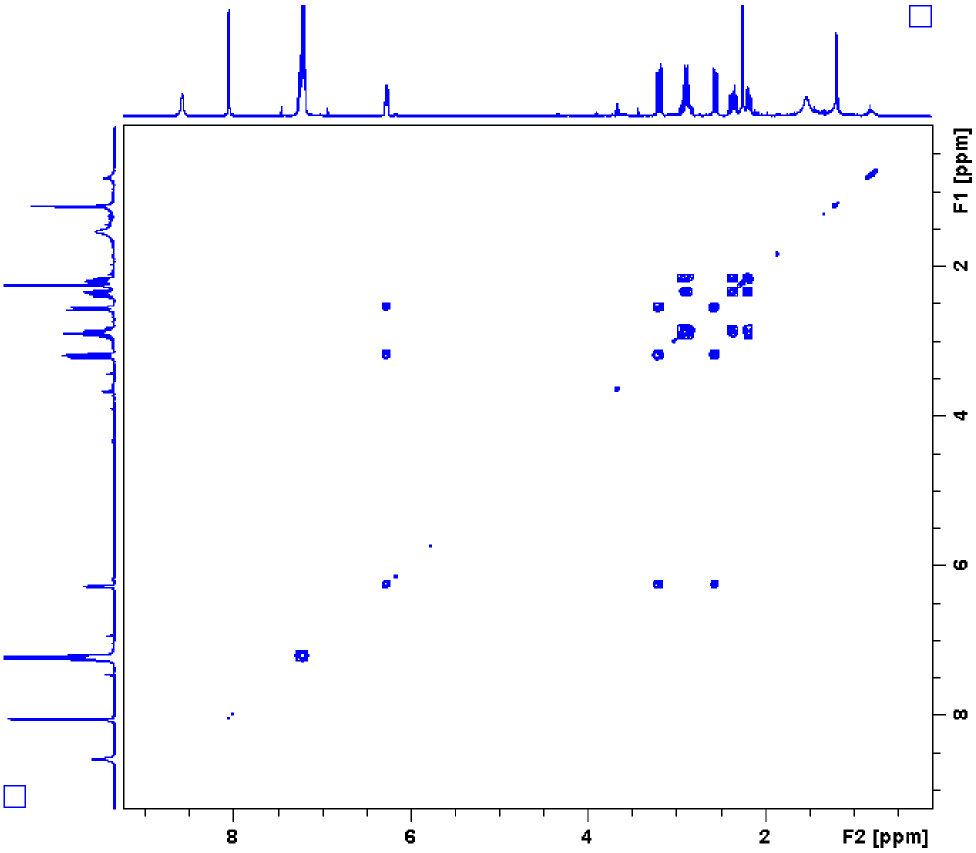


## HSQC NMR

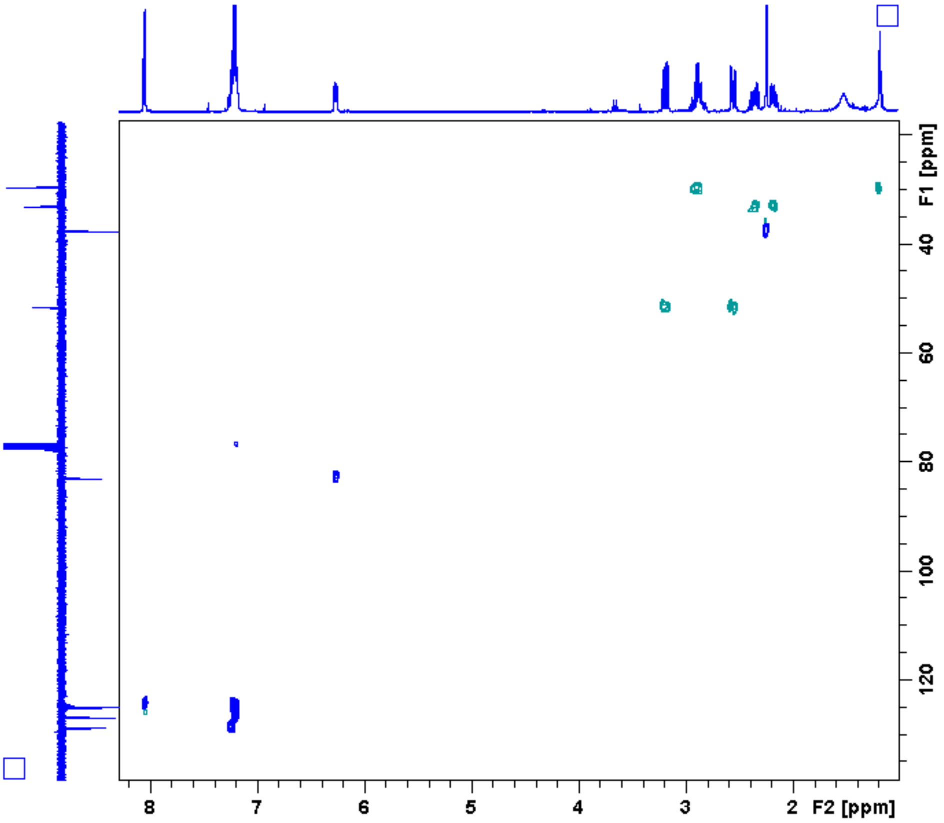

## HMBC NMR

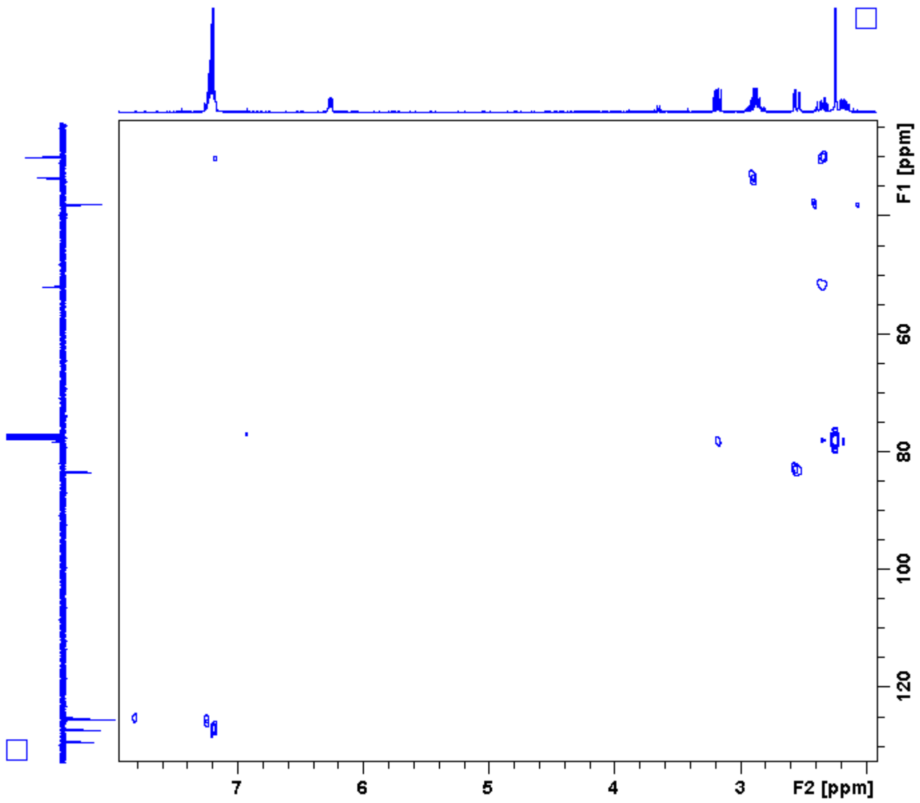


## NOESY NMR

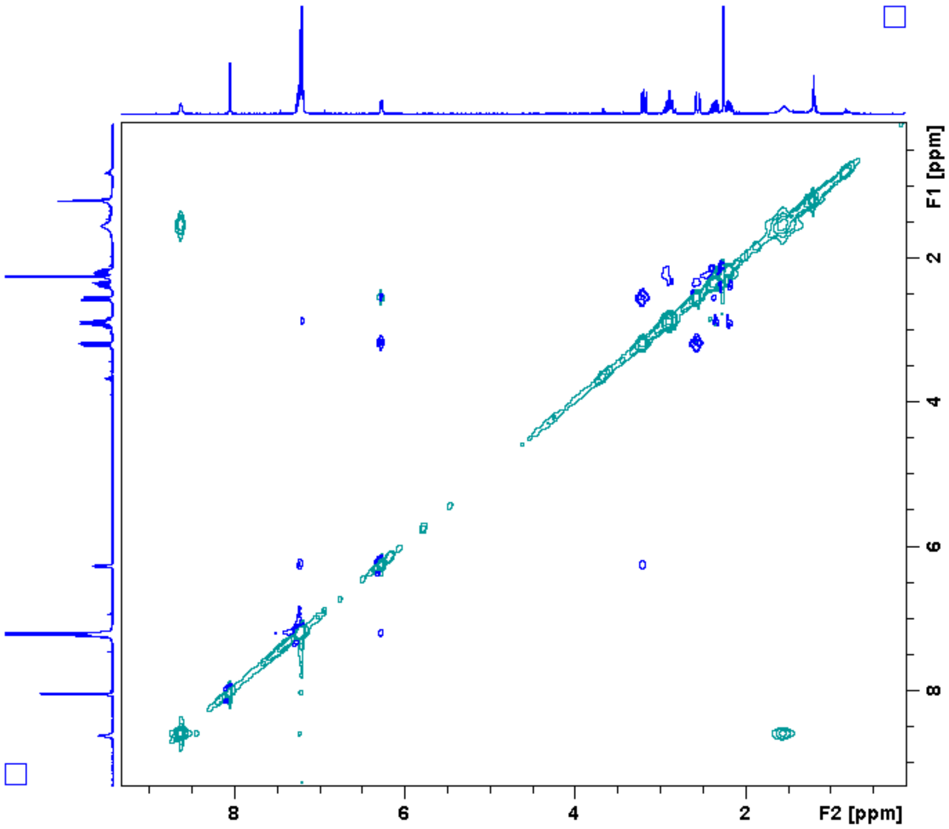

**ESI(+)-MS**

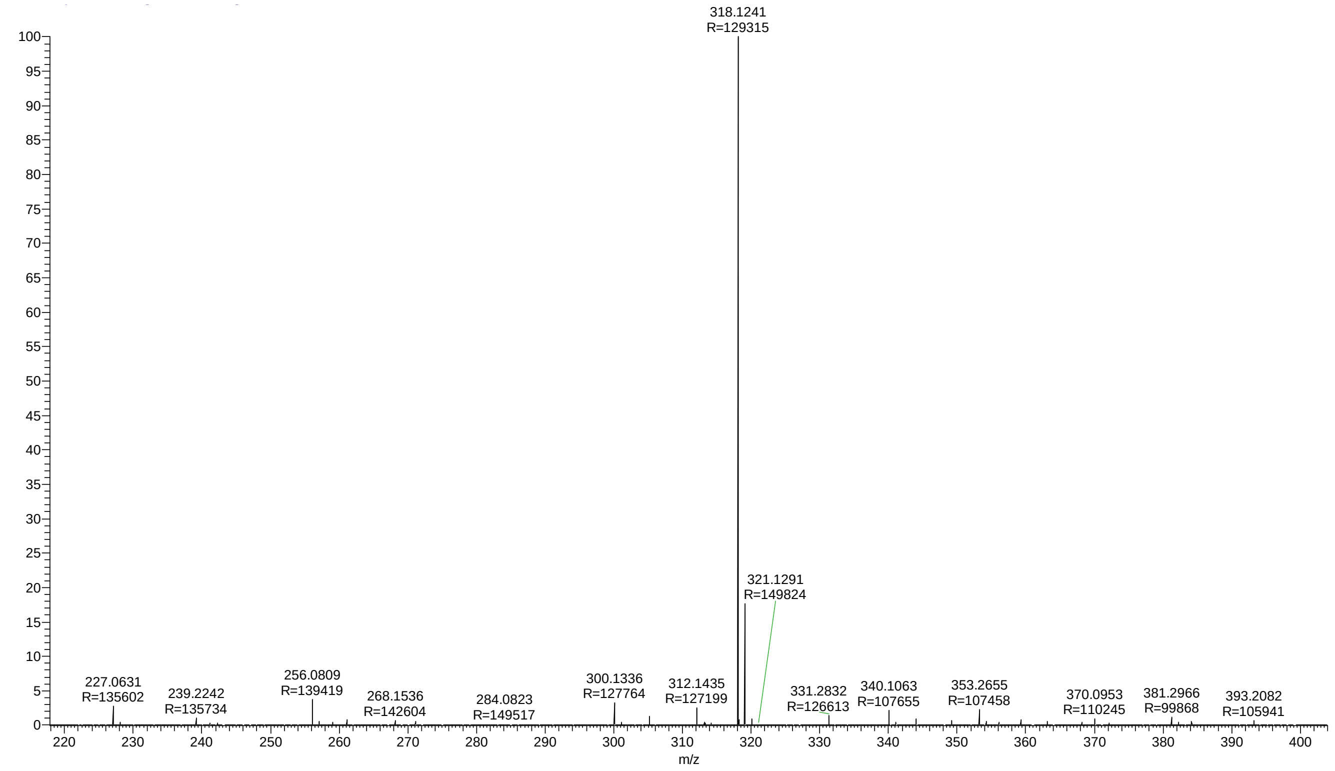

318.1241
R=129315
321.1291
R=149824
227.0631
R=135602
239.2242
R=135734
256.0809
R=139419
268.1536
R=142604
284.0823
R=149517
300.1336
R=127764
312.1435
R=127199
331.2832
R=126613
340.1063
R=107655
353.2655
R=107458
370.0953
R=110245
381.2966
R=99868
393.2082
R=105941
m/z

## *Exo*-5’-Adenyl-2’-methyl-spiro-[indane-3,3’-isoaxazolidine]. (*Exo*-5d)

### $^{1}$H NMR

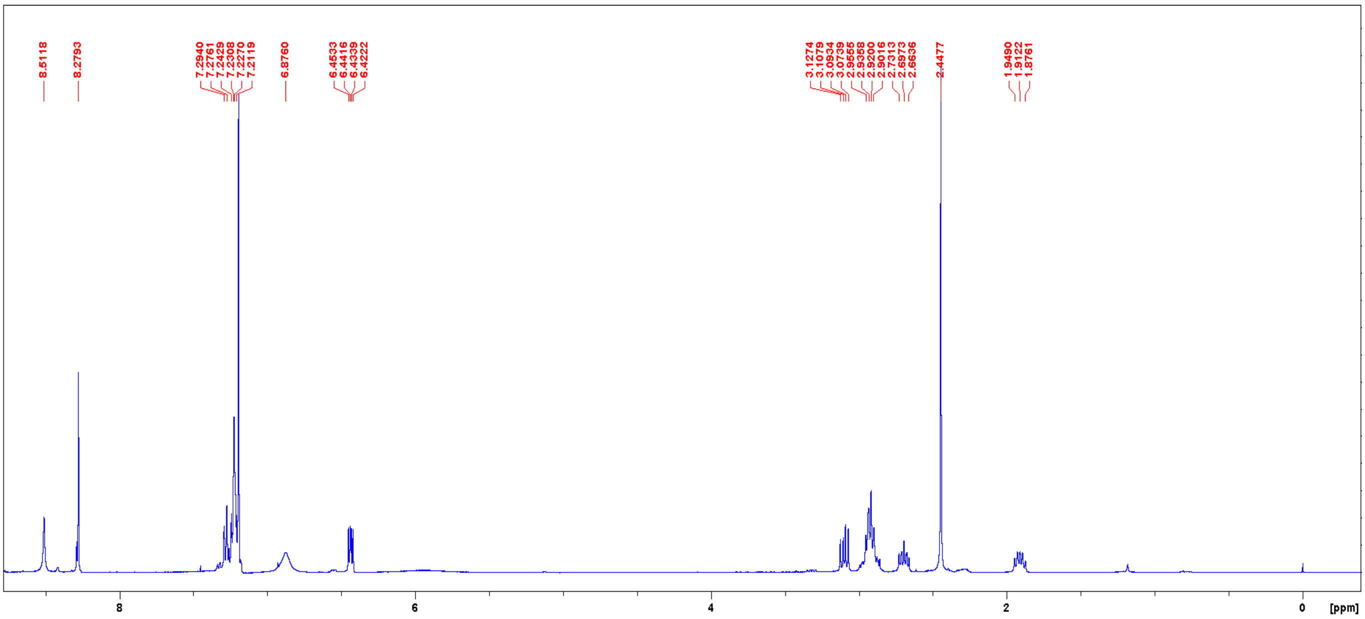


### $^{13}$C NMR

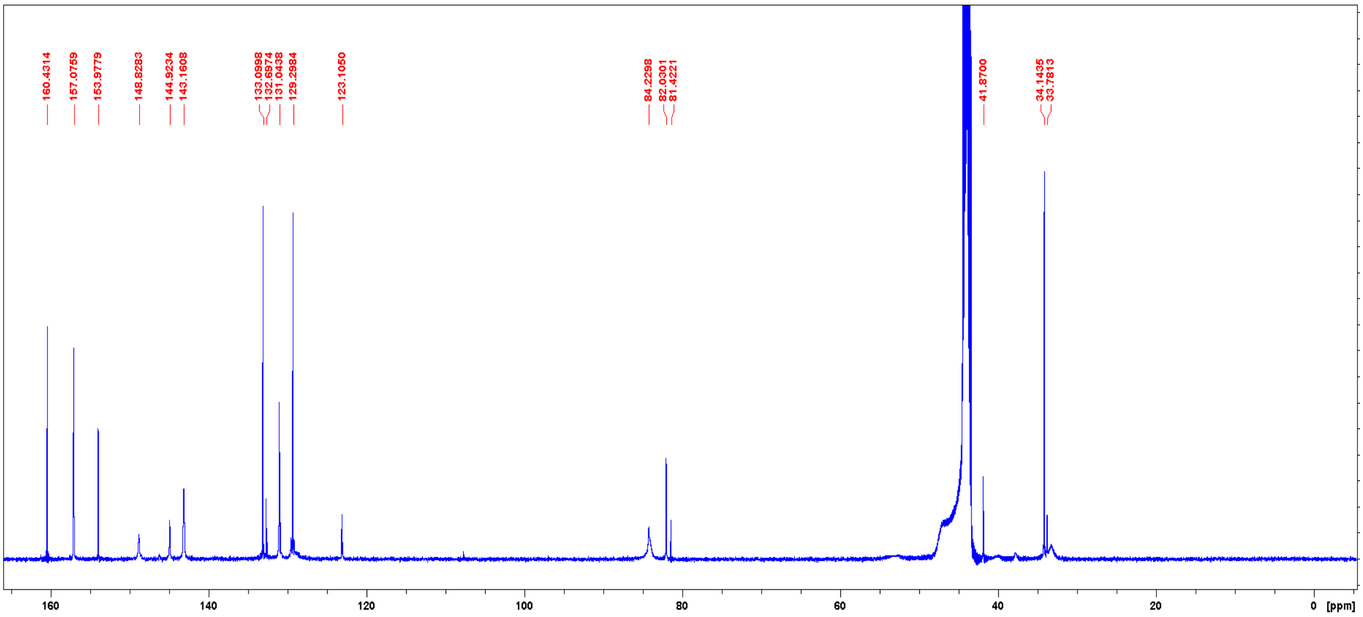

## COSY NMR

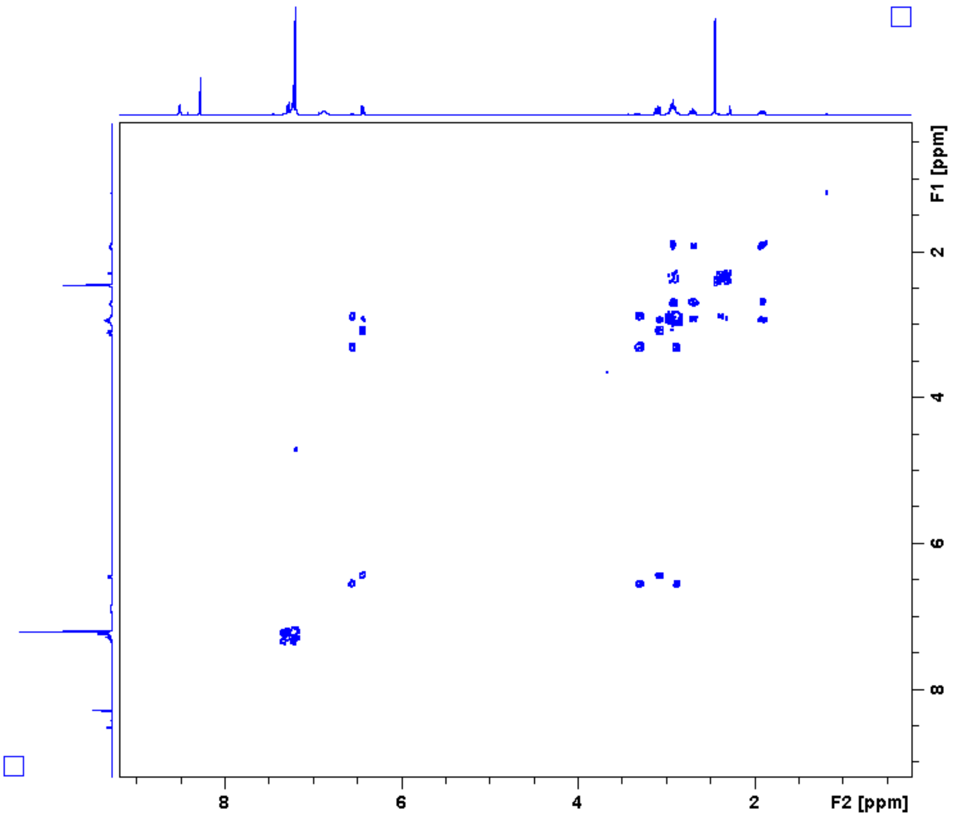


## HSQC NMR

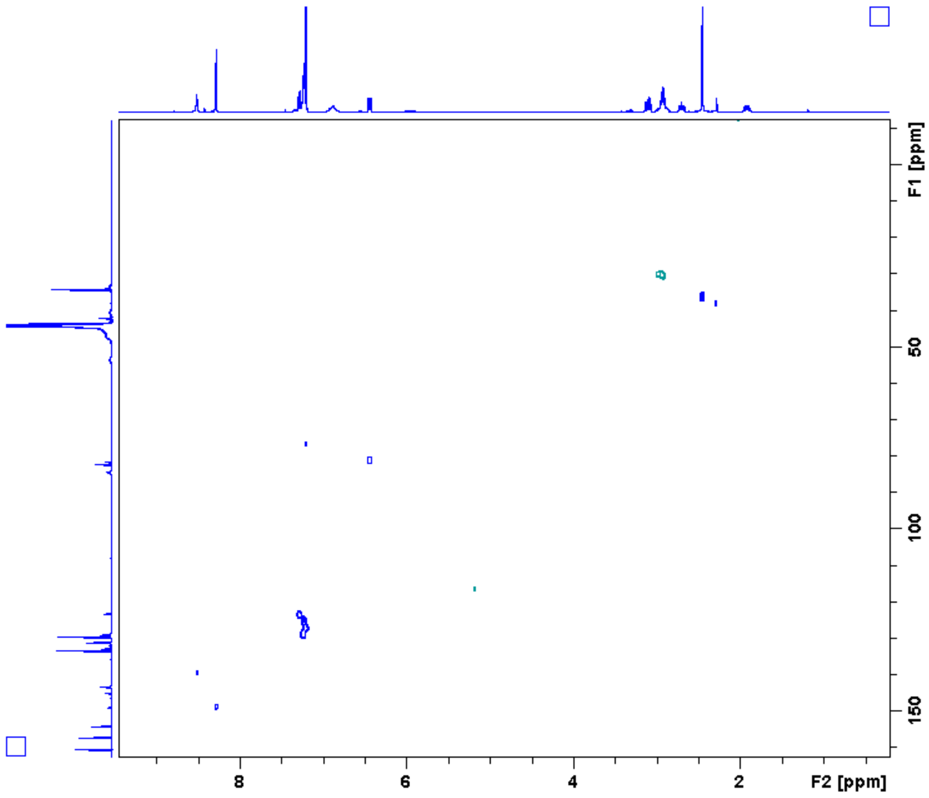

## NOESY NMR

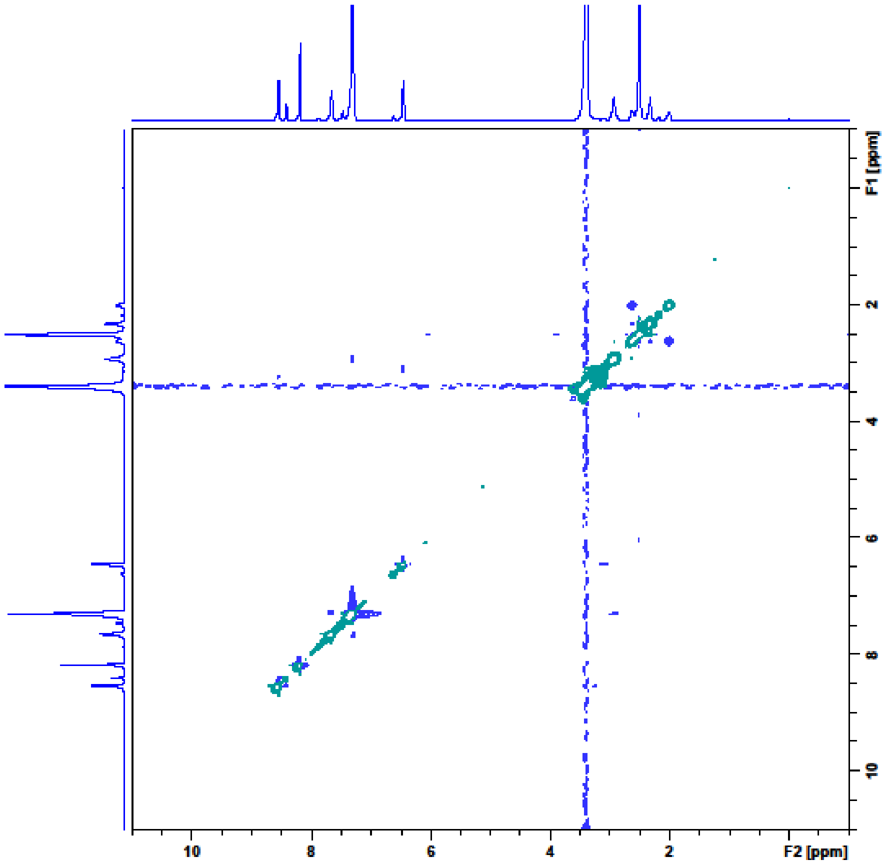


## ESI(+)-MS

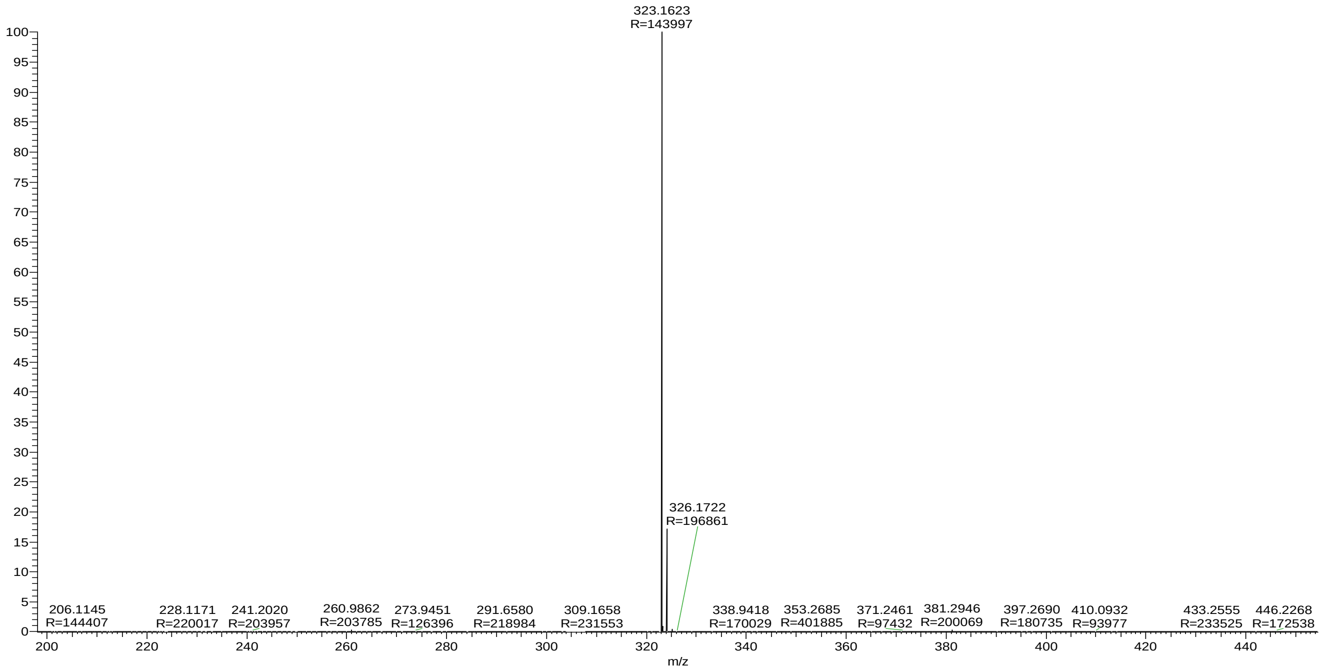

## Biological Assays

**Table S1**. Control of cell growth (%) of compounds ***Exo*-5a, *Exo*-5b**, ***Exo*-5c**, ***Endo*-5c** and ***Exo*-5d** against A549 at the concentration 25, 50, 100, 200, 250 µM.

| **Concentration** | ***Exo*-5a** | ***Exo*-5b** | ***Exo*-5c** | ***Endo*-5c** | ***Exo*-5d** |
|---|---|---|---|---|---|
| | | | | | |
| 250 µM | 33.42745±0.79 | 49.12845±1.32 | 45.23854±1.06 | 48.12845±0.14 | 47.12895±1.09 |
| 200 µM | 53.13453±0.57 | 62.26452±0.98 | 57.19457±0.61 | 60.12653±0.29 | 63.93083±1.09 |
| 100 µM | 76.36228±1.09 | 85.11892±0.68 | 80.08248±0.18 | 83.09462±0.50 | 87.80206±0.87 |
| 50 µM | 81.55023±0.57 | 91.27494±0.98 | 85.91349±1.01 | 95.17278±0.29 | 95.73523±0.49 |
| 25 µM | 84.63976±0.86 | 97.41024±1.03 | 89.53971±0.92 | 97.05963±0.81 | 98.60073±0.16 |
| Assay SRB: Data expressed as means ±SD of three independent observations. $^{*}p < 0.05$. | | | | | |

**Table S2.** Control of cell growth (%) of compound ***Exo*-5a** against human SH-SY5Y neuroblastoma cell line at the concentration 25, 50, 100, 200, 250 µM.

| **Concentration** | ***Exo*-5a** |
|---|---|
| 250 µM | 20.15284±0.34 |
| 200 µM | 38.29651±1.00 |
| 100 µM | 52.07534±0.59 |
| 50 µM | 64.06154±1.00 |
| 25 µM | 80.69501±0.19 |
| Assay SRB: Data expressed as means ±SD of three independent observations. $^{*}p < 0.05$. | |

## Computational Studies

### *Molecular docking results*

**Table S3.** Result of Docking score of input ligands to MDM2.

| Ligand | Docking score (Kcal/mol) |
|---|---|
| 5c (3S,5R) | -4.890 |
| 5c (3S,5S) | -4.828 |
| 5d (3S,5S) | -4.818 |
| 5c (3S,5S) | -4.780 |
| 5c (3S,5R) | -4.774 |
| 5a (3R,5R) | -4.737 |
| 5c (3R,5R) | -4.532 |
| 5b (3S,5S) | -4.454 |
| 5a (3S,5S) | -4.428 |
| 5a (3R,5S) | -4.424 |
| 5b (3R,5R) | -4.344 |
| 5b (3S,5S) | -4.338 |
| 5c (3R,5S) | -4.203 |
| 5a (3S,5R) | -4.160 |
| 5b (3S,5R) | -4.151 |
| 5a (3S,5R) | -4.110 |
| 5b (3S,5R) | -4.092 |
| 5c (3S,5R) | -4.081 |
| 5b (3R,5S) | -3.990 |
| 5b (3R,5R) | -3.975 |
| 5d (3R,5R) | -3.971 |
| 5a (3R,5R) | -3.970 |
| 5d (3R,5S) | -3.969 |
| 5d (3R,5R) | -3.742 |
| 5a (3R,5R) | -3.685 |
| 5d (3S,5R) | -3.671 |
| 5d (3S,5R) | -3.602 |
| 5b (3S,5R) | -3.585 |
| 5d (3R,5S) | -3.546 |
| 5d (3S,5R) | -3.495 |
| 5c (3S,5S) | -3.491 |
| 5d (3R,5S) | -3.468 |
| 5d (3R,5R) | -3.414 |
| 5c (3R,5R) | -3.410 |
| 5d (3S,5R) | -3.400 |
| 5a (3R,5R) | -3.298 |
| 5d (3S,5S) | -3.249 |

| | |
|---|---|
| 5c (3R,5R) | -3.231 |
| 5c (3R,5R) | -3.226 |
| 5c (3S,5S) | -3.140 |
| 5c (3S,5R) | -3.063 |
| 5b (3S,5S) | -2.899 |
| 5d (3S,5R) | -2.360 |

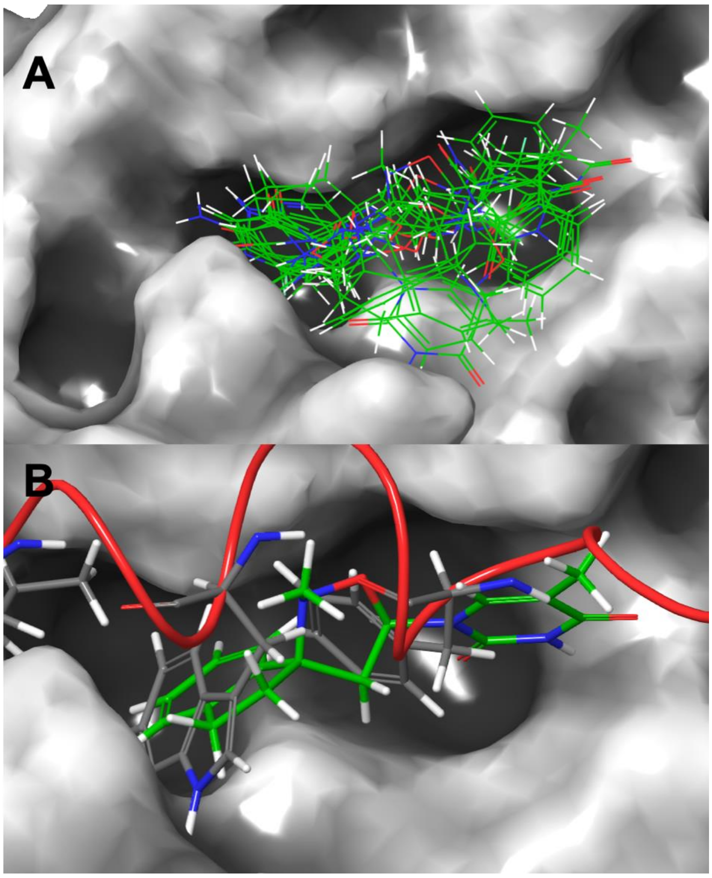


**Figure S1.** Stacked plot of minimum energy docking poses for **5a-5d** derivatives (A). Detail of ***exo-(3R,5R)*-5a** in green superimposed to p53 in red and grey (B).

*Molecular Dynamic Simulations*

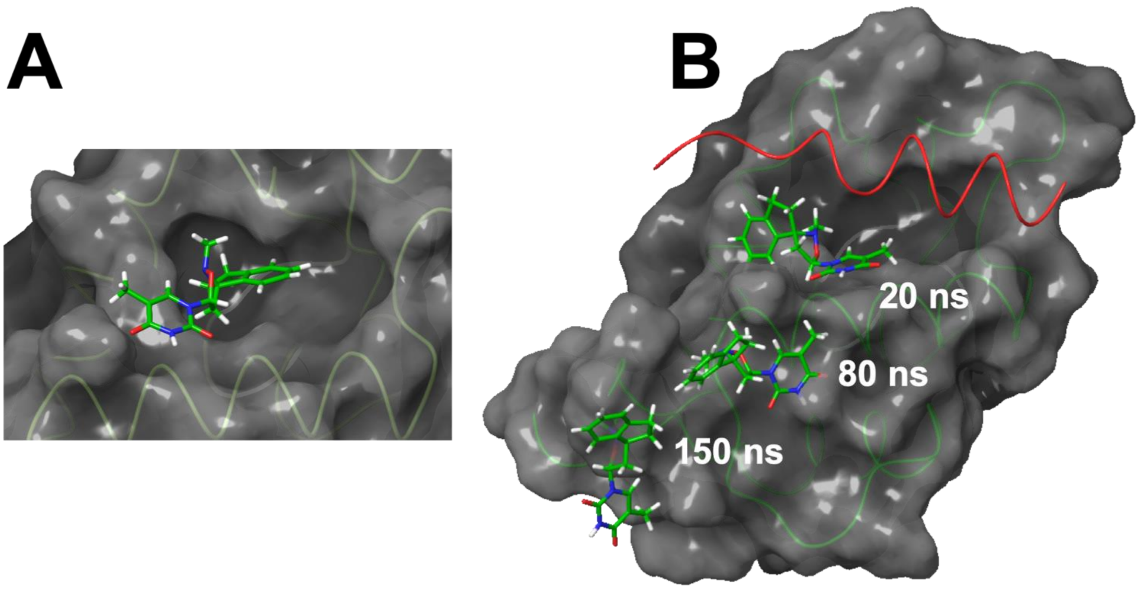


**Figure S2.** Snapshot from MD simulation of ***exo-(3R,5R)*-5a** bounded to MDM2 (A). Snapshots from MD at different simulation times of ***endo-(3R,5S)*-5a** bounded to complex MDM2-p53 (B).

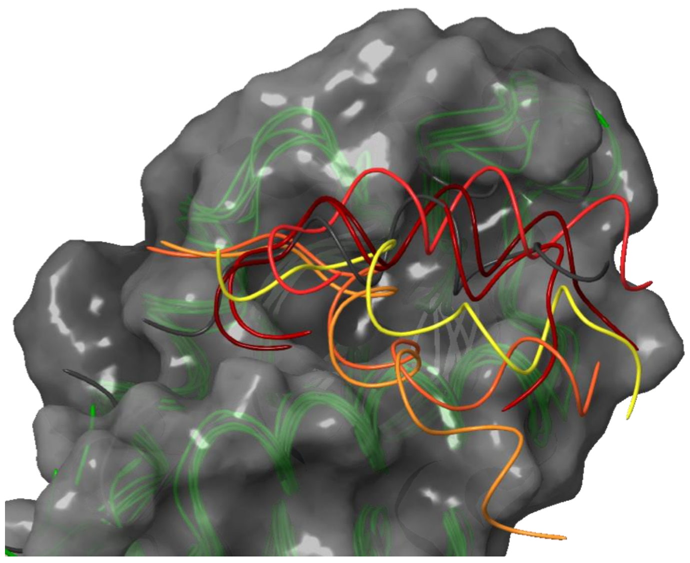

**Figure S3.** Snapshots from MD simulation of unbounded p53 and MDM2 at different simulation times. MDM2 in green, p53: starting structure (yellow), 100 ns (light orange), 200 ns (orange), 300 ns (light red), 400 ns (red), 500 ns (dark red).
